# Topochemical Photopolymerization above the Bulk Melting Temperature in Noncovalent Monolayers on Graphite

**DOI:** 10.1002/smll.202509179

**Published:** 2025-09-18

**Authors:** Joseph A. Garfield, Tobiloba E. Awoyemi, Emmanuel K. Nava, Shelley A. Claridge

**Affiliations:** ^1^ Department of Chemistry Purdue University 560 Oval Drive West Lafayette IN 47904 USA; ^2^ Weldon School of Biomedical Engineering Purdue University 206 S Martin Jischke Dr. West Lafayette IN 47907 USA

**Keywords:** monolayers, polymerization, scanning probe microscopy, surface chemistry, 2D materials

## Abstract

While studies of surface‐templated topochemical reactions often focus on the central role of monomer ordering, the reactions themselves frequently require Ångström‐scale dynamics to form new bonds. Here, it is showed that elevated temperatures increase the topochemical reaction efficiency of 10,12‐tricosadiynoic acid (TCDA), a widely utilized commercial diacetylene monomer, assembled on highly oriented graphite (HOPG) substrates. Up to ≈45 °C, Arrhenius temperature dependence is observed for the reaction, with E_a_ = 5.9 kcal mol^−1^ (0.26 eV), consistent with limitations imposed by the propagation step of the reaction, which requires monomer dynamics within the lattice. At higher temperatures, t_0.5_ does not continue to decrease. However, the number average degree of polymerization continues to increase, from 97 at 5 °C to 248 at 65 °C, and the number density of polymers formed per incident photon increases by 6–13‐fold at elevated temperatures (45–65 °C) in comparison with polymerization at 5 °C. Together, these changes in the on‐surface reaction greatly increase molecular sheet integrity, resulting in a 10‐fold increase in the efficiency of a covalent mesh‐forming reaction that transfers TCDA sheets to soft polydimethylsiloxane.

## Graphical abstract

Photopolymerizing monolayers of diacetylene monomers noncovalently adsorbed to graphite above the bulk melting point of the monomer greatly increases the efficiency of the on‐surface topochemical photopolymerization reaction.

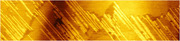

## 1 Introduction

Surface‐templated topochemical reactions^[^ ^1^ ^]^ represent a powerful strategy for generating molecular network architectures for new materials, leveraging pre‐ordering of reactants on a substrate to promote chemical crosslinking. A longstanding challenge in topochemical polymerization is that efficient reactions rely on strong molecular ordering to afford regular placement of the reactive groups, but the reactions themselves typically also require Ångström‐scale molecular dynamics.^[^ ^2^ ^]^ Such dynamics can be restricted by both the close placement of adjacent monomers and strong molecule‒substrate interactions. Thus, strategies that enable highly ordered monomers to undergo complete, rapid conversion to polymer, maximizing propagation lengths and minimizing defect density, are of increasing importance.

Diacetylene (DA) polymerization reactions exemplify the need to balance ordering and dynamics for topochemical reactions. Reactivity of DA crystals has been widely studied since the late 1960s,^[^ ^3^ ^]^ utilizing a broad range of monomers, although polymerization mechanisms and kinetics have typically been examined in 3D crystals of simple small molecules such as 2,4‐hexadiyne‐1,6‐diol‐bis‐(*p*‐toluenesulfonate).^[^ ^4^ ^]^ Initiation involves formation of a diradical;^[^ ^5^ ^]^ the subsequent propagation reaction with adjacent monomers is facilitated by minimal distances (≈4 Å) between bond‐forming carbons,^[^ ^6^ ^]^ associated with highly ordered monomers. However, rehybridization to form the polydiacetylene (PDA) alters lattice parameters slightly,^[^ ^6^ ^]^ meaning that monomer ordering must be balanced against the mobility required for the propagation step of polymerization. For maximally efficient DA photopolymerizations, E_a_ is typically ≈0.1 eV,^[^ ^7^ ^]^ with average degrees of polymerization (DP) in the range from 1000–2000. For molecules in which molecular packing restricts the propagation step, higher E_a_ values (0.2–0.3 eV) have been reported, along with lower DP values.^[^ ^7^ ^]^

**Scheme 1:**
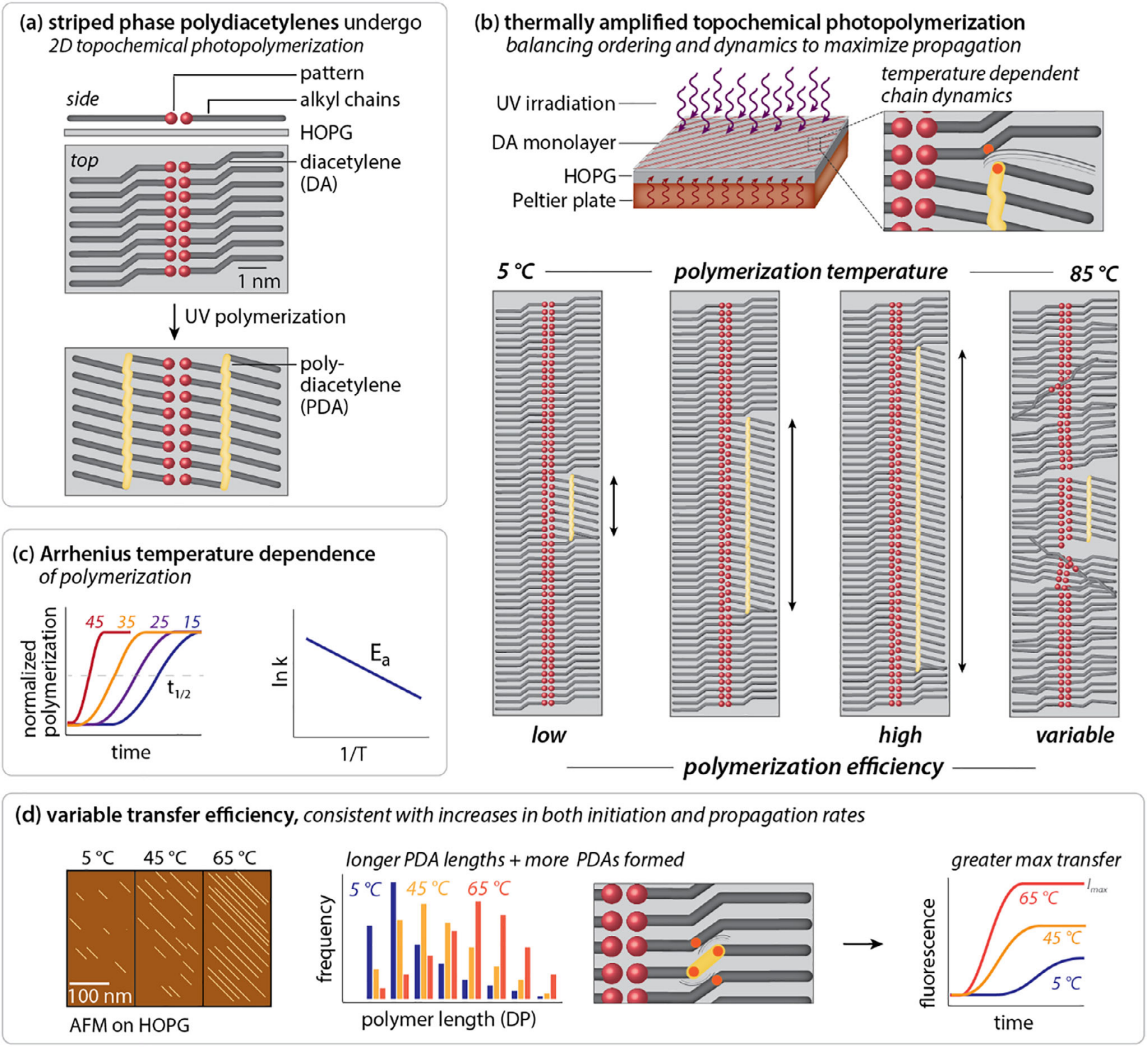
Schematic representations of a) striped phase structure and photopolymerization, b) impacts of temperature on propagation and polymer length, c) Arrhenius temperature dependence of TCDA polymerization, and d) impacts of propagation rate on PDA polymer length and transfer efficiency.

DA monomers with long alkyl chains and/or aromatic rings can also assemble into ordered molecular layers on highly oriented pyrolytic graphite (HOPG).^[^ ^1^, ^8^ ^]^ One of the most common monomers of this type, 10,12‐tricosadiynoic acid (TCDA), forms layers with the alkyl chains lying parallel to the HOPG, generating lamellar molecular layers ≈0.3 nm thick, comprising 1‐nm resolution chemical patterns with a sub‐10‐nm pitch (**Scheme** **1**a, left). Irradiation of the molecular layer generates arrays of precisely spaced PDAs (Scheme 1a, right). In contrast with polymerization in a 3D crystal, polymerization in this system requires molecular dynamics within the 2D adsorbed layer, as well as against relatively strong alkyl‒π interactions with the HOPG. Previously, we showed that monomers that experience stronger molecule‒molecule and molecule‒substrate interactions typically exhibit shorter propagation lengths,^[^ ^9^ ^]^ limiting their efficacy in a subsequent 2D mesh‐forming process that relies on relatively high PDA DPs (100–200 repeat units).^[^ ^9^, ^10^ ^]^

Most on‐surface topochemical photopolymerization (OSTP) reactions are carried out without heating, promoting molecular ordering. However, we questioned whether elevated temperatures might increase polymerization efficiency by promoting monomer dynamics (Scheme 1b). The noncovalent alkyl‒HOPG contact might appear to limit the useful range of temperature stability for thermally accelerated topochemical photopolymerization. However, prior differential scanning calorimetry of simple alkanes in expanded graphite suggested that the disorder temperature of the molecular layer directly adsorbed to the graphite can be as much as 40–50 °C above the equivalent bulk melting point.^[^ ^11^ ^]^ While disorder transition temperatures might be different for adsorbed molecular layers in the absence of additional molecular layers, it would be reasonable to expect stability well above room temperature.

Although OSTP reactions of alkyl diacetylenes on HOPG have been carried out since at least 1997,^[^ ^12^ ^]^ the majority of the early work was carried out with a focus on molecular electronics,^[^ ^8^, ^12^, ^13^ ^]^ typically using single‐polymer scanning probe characterization at low conversion. The only temperature‐dependent kinetics to date are for 10,12‐pentacosadiyn‐1‐ol, from 8–28 °C, which indicated E_a_ ≈8 kcal mol^−1^ in the initial lag phase of polymerization (<10% conversion to polymer), based on scanning tunneling microscopy measurements of individual polymer lengths.^[^ ^14^ ^]^ Prior work has highlighted the importance of alkyl chain ordering (achieved through either selection of appropriate ordered phase, or thermal annealing),^[^ ^14^, ^15^ ^]^ as well as the role of oxygen in terminating polymerization.^[^ ^15^, ^16^ ^]^

To understand whether OSTP carried out at controlled elevated temperatures could be used to increase the efficiency of this class of on‐surface reactions, we fabricated a temperature‐controlled stage and environmental chamber. These were used to examine the Arrhenius temperature dependence of the OSTP reaction of TCDA (Scheme 1c), as measured through both populations of individual polymer lengths measured by atomic force microscopy (AFM) and by the efficiency of a mesh‐forming reaction that transfers the molecular sheet to polydimethylsiloxane (PDMS).^[^ ^9^, ^17^ ^]^ Overall, we observed that polymerization and transfer efficiency increase for temperatures up to 65 °C. At higher temperatures (Scheme 1d), efficiency benchmarks become more variable, consistent with impacts from partial alkyl chain disordering (Scheme 1b). Overall, our findings point to a broadly generalizable strategy for controlling topochemical reaction efficiency independent of monomer structure, to create highly crosslinked molecular sheets with embedded nanoscale functional patterns.

## 2 Results and Discussion

### 2.1 Predicting Monolayer Stability Through Molecular Dynamics

**Figure 1:**
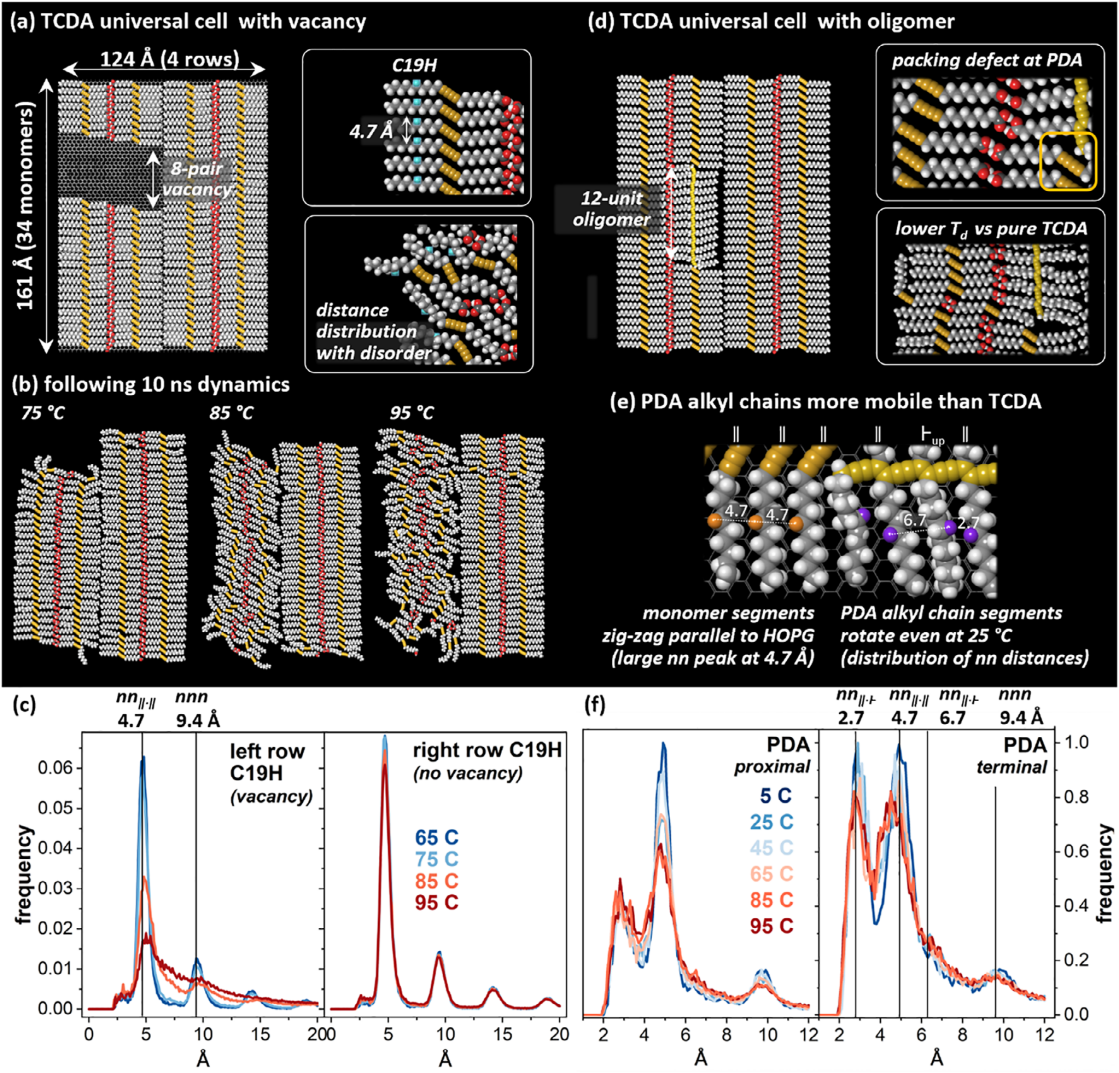
a) Universal cell for TCDA monolayer 4 × 34 monomers in dimension, with a 2 × 8 set of monomers removed, used as a repeating unit for molecular dynamics simulations. Insets illustrate the set of hydrogen atoms appended to alkyl carbon 19 of each molecule, which were used to track molecular disordering at elevated temperature. b) Snapshots of the model from (a) after 10 ns of dynamics at 75, 85, or 95 °C. c) RDFs of distances between C19 hydrogens in either a vacancy row (left) or a tightly packed row (right), following dynamics at 65 °C (dark blue), 75 °C (light blue), 85 °C (orange), or 95 °C (red). d) Molecular model of TCDA monolayer 4 × 34 monomers in dimension, containing a 12‐unit polymerized oligomer in the second row. Insets illustrate small packing defects created around the PDA, and impacts observed on disordering transitions in comparison with fully‐packed TCDA models. e) Model illustrating alkyl chain rotational dynamics observed predominantly for PDA chain segments, across the entire temperature range tested. f) Radial distribution functions for PDA monomer H atoms at temperatures from 5 to 95 °C.

We simulated dynamics of unpolymerized and polymerized monolayers at temperatures from 5–165 °C, in order to establish reasonable parameters for experimental studies. Elevated temperatures are frequently associated with more rapid reactions, but also with molecular disordering processes, which would decrease the efficiency of OSTP reactions. We evaluated molecular dynamics (MD) trajectories of both full and defective unpolymerized monolayers and monolayers with an embedded oligomer (**Figure** **1**). In contrast with smaller‐scale models we have used previously, in this study, molecular dynamics simulations were carried out with periodic boundary conditions that enabled the system to more closely mimic the behavior of an extended monolayer (Figure S17, Supporting Information, provides more details of the process for establishing universal cell structure, including molecular packing density and boundary conditions). Full monolayers of unpolymerized TCDA remained stable at temperatures up to 115 °C in MD simulations up to 5 ns, although segments of molecules exhibited increasing frequency and range of dynamics at higher temperatures (Figures S18–S21, Supporting Information).

Molecular dynamics simulations of perfect crystals typically overestimate experimental melting temperatures.^[^ ^18^ ^]^ A variety of strategies have been developed to address this discrepancy for atomic and small molecular systems,^[^ ^18^, ^19^ ^]^ including the addition of voids.^[^ ^20^ ^]^ For the system studied here, under experimental conditions, HOPG step edges and domain boundaries, as well as internal molecular packing defects, would be expected to locally decrease the temperature required for disordering. Since it was not straightforward to model a domain boundary directly within the cell size that was feasible to simulate, we introduced a 16‐molecule (8‐dimer) vacancy (void) spanning two of the four molecular rows (Figure 1a). In such models, all 4 rows remained ordered after 10 ns dynamics at 65 or 75 °C. At 85 or 95 °C, defective rows disordered, but the tightly packed molecular rows remained ordered. These effects are visible in the models in Figure 1b, as well as in RDFs quantifying the nearest‐neighbor (nn) distances for hydrogens bound to carbon 19 in each molecule (acquired from timepoints in the 5–10 ns interval within the simulation). In Figure 1c, the nn peak at 4.7 Å is sharp at 65 and 75 °C (dark and light blue traces, respectively), but decreases in intensity at 85 °C (orange trace) and further decreases at 95 °C (red trace). For the tightly packed pair of molecular rows shown in the right panel of Figure 1c, the peak structure remains consistent in the 5‐ns window comprising the RDF.

Because the aim of the experiments below is to polymerize the entire monolayer, we also examined the impacts of polymerization on dynamics and disordering, using a model that included a 12‐unit oligomer (Figure 1d). Inclusion of a PDA in the lattice creates small packing defects at the polymer ends after reminimization; at higher temperatures, these facilitated slip defect formation (Figure S21, Supporting Information), although in the model tested, disordering began at 105 °C, well above the melting point of the void model.

We also examined the dynamics of the PDA in comparison with the surrounding monomers below the melting temperature. Previously, experiments by others have illustrated that, well below the solid‒liquid transition temperature, simple straight‐chain alkanes assembled on HOPG undergo a solid‒solid transition to a rotator phase with expanded chain spacing,^[^ ^11^ ^]^ which might be expected to impact propagation rates during polymerization.

When alkyl chain segments zig‐zag parallel to the HOPG, C19 hydrogens (labeled in orange near the left edge of Figure 1e) exhibit nearest neighbor spacings (nn_‖∙‖_) ≈4.7 Å, with a next nearest neighbor (nnn) peak ≈9.4 Å, as seen in Figure 1c. Rotation of an alkyl chain segment so that its alkyl chain zig‐zags perpendicular to the HOPG produces a RDF new peak (nn_‖∙Ͱ_) at ≈2.9 Å, and a second nn_‖∙Ͱ_ peak at ≈6.7 Å (Figure 1f). Notably, PDA alkyl chain segments exhibited substantial rotation and associated dynamics across the simulated temperature range, while monomer alkyl chains did not, suggesting that the PDA may dominate the dynamics associated with propagation.

### 2.2 Assembly of sPDA Monolayers on HOPG

**Figure 2:**
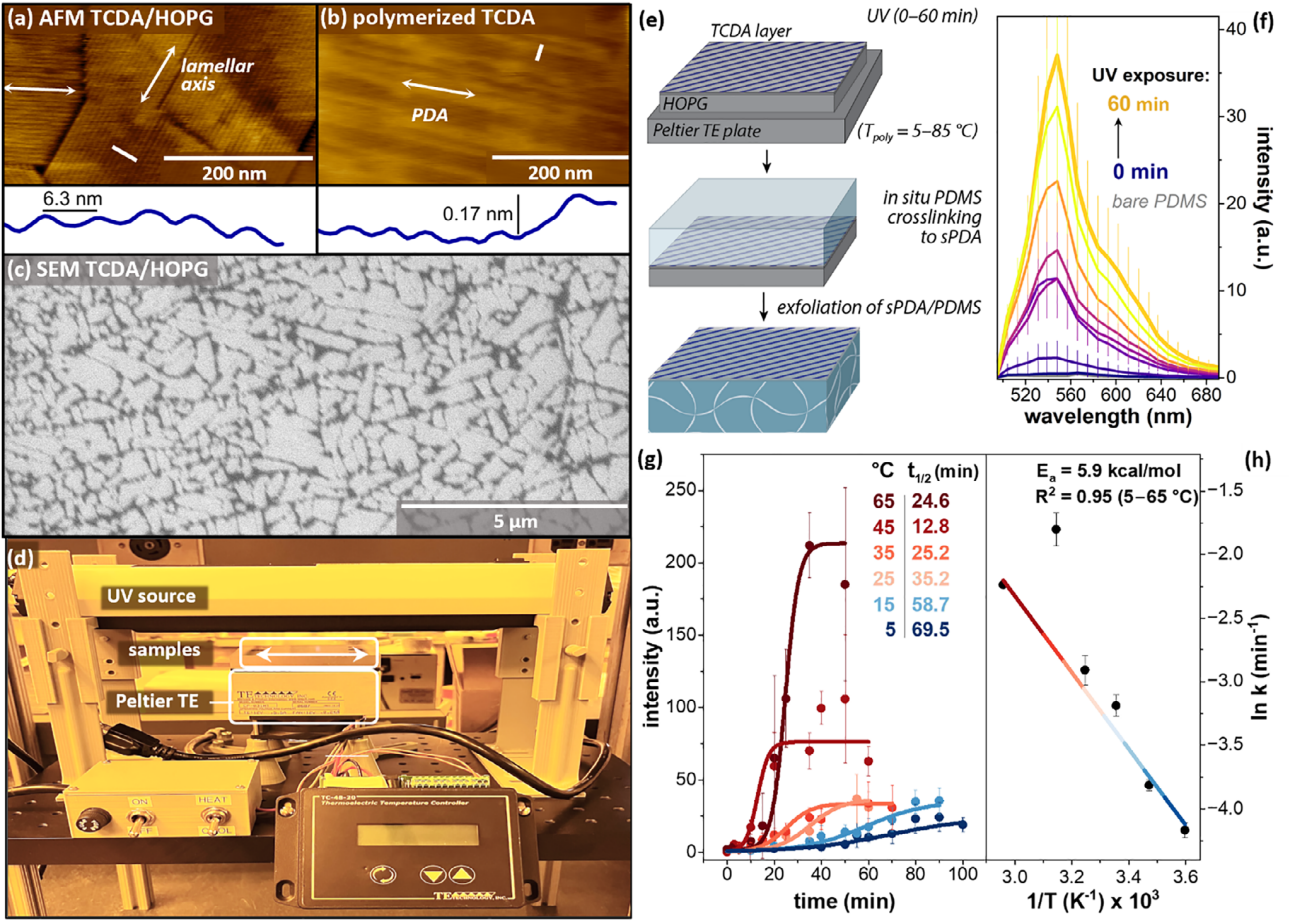
a,b) AFM images of (a) unpolymerized and (b) polymerized TCDA/HOPG; insets in (a,b) show line scans acquired in regions highlighted with white lines, measuring lamellar periodicity and height of protruding PDA backbone, respectively. c) SEM image of domain structures of polymerized TCDA/HOPG. d) Peltier thermoelectric (TE) assembly, consisting of UV source and positioning arms, Peltier TE plate, and TE temperature controller, housed in N_2_ environmental chamber. e) Schematic illustrating temperature‐controlled polymerization of TCDA on HOPG, followed by crosslinking to PDMS and exfoliation onto PDMS, where transferred sPDA is characterized by fluorescence emission. f) Fluorescence emission spectra of sPDAs transferred to PDMS after photopolymerization times of 0 min (purple) to 60 min (gold) on HOPG at a setpoint temperature of 25 °C. g) Time‐dependent conversion of TCDA to sPDA based on quantification of peak emission intensities similar to those shown in panel f), with polymerization temperatures from 5 °C (dark blue) to 65 °C (dark red). h) Arrhenius plot based on t_0.5_ – t_0.1_ values from g).

To examine the temperature‐dependence of DA striped phase polymerization experimentally, we prepared striped phase monolayers of TCDA on HOPG (**Figure** **2**). Assembly of monolayers was performed using Langmuir–Schaefer (LS) transfer,^[^ ^21^ ^]^ in which amphiphiles are first deposited on an aqueous subphase and compressed to produce a layer of standing phase amphiphiles. An HOPG substrate at a moderately elevated temperature is then slowly lowered into contact with the Langmuir film, causing some of the molecules in the standing phase film to be converted to the striped phase. Our prior work has demonstrated the efficacy of LS conversion in preparing striped phases exhibiting large ordered molecular domains and uniform surface coverage,^[^ ^21^, ^22^ ^]^ both of which are important for applications that utilize the monolayer chemistry for function. Following LS transfer, monolayers undergo UV‐initiated photopolymerization (λ_exc_ = 254 nm). See Experimental Methods in the Supporting Information for a more detailed discussion of monolayer preparation and polymerization.

In AFM images of TCDA monolayers prepared as described above, the lamellar width is ≈6.3 nm unpolymerized (Figure 2a), matching dimensions illustrated by theoretical models (*e.g*., those in Figure 1). Following exposure to UV light, individual polymerization events are visible due to a conformational change that produces a small topographical protrusion of the PDA backbone (≈0.17 nm) in AFM images (Figure 2b), which are used in the experiments described below to evaluate polymer length and extent of polymerization of the monolayer. Microscale molecular domain structure on HOPG can be observed in SEM images (Figure 2c), as we have shown previously.^[^ ^22a^ ^]^

### 2.3 Evaluating Arrhenius Temperature Dependence of Polymerization

To elucidate temperature dependence of the polymerization kinetics, we designed and constructed a Peltier thermoelectric (TE) plate assembly (Figure 2d, see Supporting Information for more detailed description), that enabled us to heat or cool samples to a desired polymerization temperature, while exposing them to a controlled UV photon flux (here, ≈2 photons nm^−2^ s^−1^), in an N_2_ environmental chamber (r.h. ≤10%, minimal O_2_) to limit termination of polymerization. UV exposure typically increased HOPG temperature by *ca*. 1 °C, with equilibrium temperature achieved within ∼1 min of turning on the UV lamp (see Figure S13, Supporting Information). All steps were carried out in a laboratory with UV‐filtered lights.

Using the Peltier TE plate, we carried out photopolymerization of TCDA at selected set point temperatures from 5–85 °C (Figure 2e), based on outputs of simulations in Figure 1. Samples were equilibrated for 30 min at the set point temperature prior to UV illumination. Samples were removed from the TE plate/UV source immediately upon reaching their designated polymerization time, and either characterized by AFM to assess individual polymer lengths, or transferred to PDMS to assess the extent of polymerization using fluorescence. Previously, we have shown that in situ crosslinking of PDMS in contact with a PDA striped phase monolayer enables the PDA to participate in the PDMS hydrosilylation (crosslinking) reaction,^[^ ^17^ ^]^ resulting in the formation of covalent bonds between PDAs in the monolayer and the PDMS mesh (Figure 2e, center). As a result, when the crosslinked PDMS is exfoliated from the HOPG, PDAs from the monolayer are also exfoliated (Figure 2e, bottom). The fraction of the monolayer that undergoes covalent transfer can be assessed by performing confocal fluorescence microscopy on the PDMS, and quantifying intensity at the PDA emission maximum, as shown in the spectra in Figure 2f. Previously, we have established that, for partially polymerized monolayers, this process provides a useful microscale method of quantifying the extent of polymerization, complementing single‐polymer quantification by AFM.^[^ ^9a^ ^]^

Here, we used this process to quantify reaction kinetics for monolayers polymerized under controlled temperature conditions for different periods of time. For samples transferred to PDMS, processing was initiated within 1 h of removal from the TE plate. PDMS curing was carried out at 35 °C for 24 h; low‐temperature curing minimizes the possibility of thermal polymerization during transfer. Monolayers exposed to these temperatures and PDMS transfer conditions in the *absence* of UV exposure produced fluorescence emission ≈1 a.u. (see Supporting Information), and are graphed as ‘0 min’ timepoints in the figures.

Reaction rates increased with polymerization temperature up to 45 °C. Quantitative comparisons for cooperative reactions with sigmoidal fits (Figure 2g) in bulk polymerizations of this type are typically made based on t_0.5_ (or t_1/2_) the value at which 50% of the maximum conversion yield is achieved (then used to calculate a rate constant using first‐order kinetics, sometimes using t_0.5_–t_0.1_),^[^ ^4b,d^ ^]^ or based on the slope in the cooperative segment of the conversion graph (e.g., from t_0.1_ to t_0.5_).^[^ ^5^, ^23^ ^]^ See the Supporting Information for further discussion regarding historical methods for calculation of rates, including the assumption of first‐order kinetics, and comparisons of the outcomes of using different methods for the experiments described here.

For photopolymerization of TCDA on HOPG, t_0.5_(5 °C) = 69.5 ± 4.0 min, decreasing to t_0.5_(45 °C) = 12.8 ± 1.6 min. An Arrhenius plot (Figure 2h) was generated, assuming first‐order kinetics and using t_0.5_ – t_0.1_ as an input to the rate equation (Figure 2g), to compare rates for the cooperative phase of the reaction.^[^ ^4d^ ^]^ This approach yielded a calculated E_a_ = 5.9 ± 0.6 kcal mol^−1^ (0.26 ± 0.03 eV) with a frequency factor (A) of 4.4 × 10^4^. Polymerization at 85 °C does not further decrease t_0.5_ (see Supporting Information).

**Figure 3:**
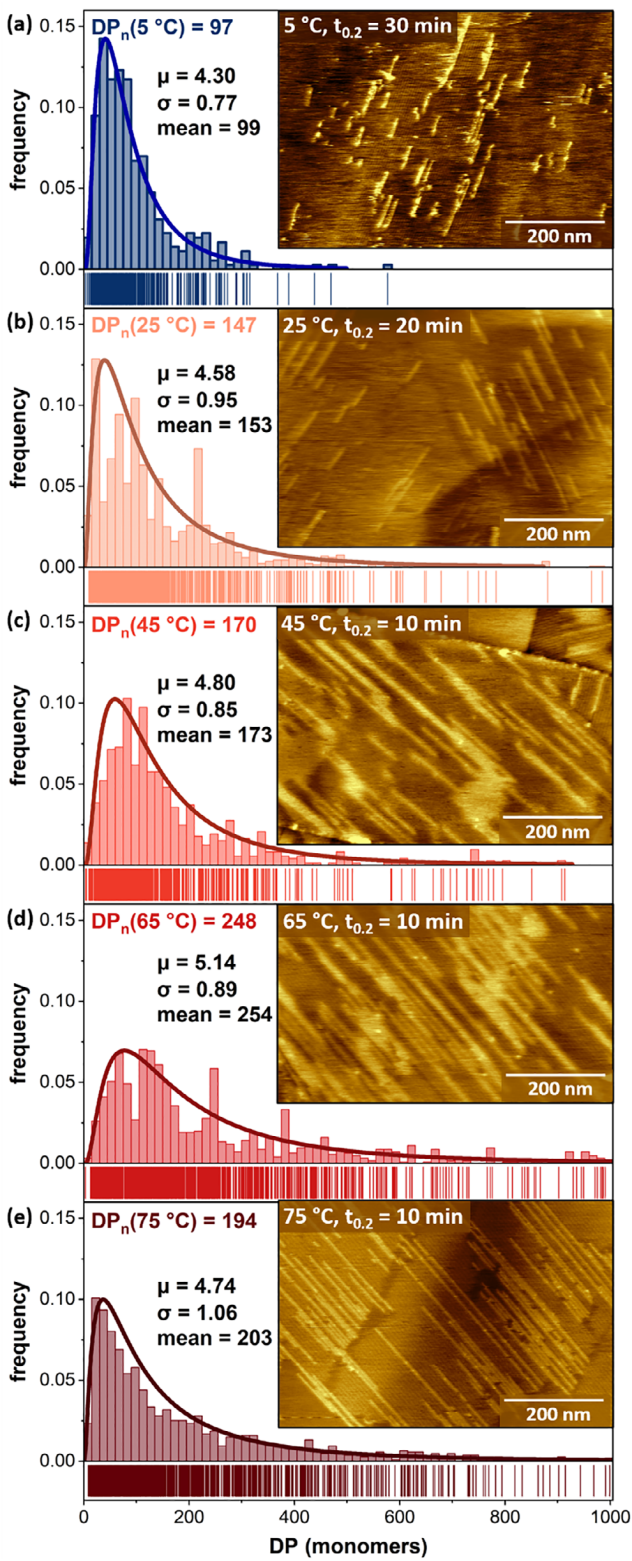
Histograms of distributions of PDA lengths used to calculate number average degree of polymerization (DP_n_) for polymerization at a) 5 °C, b) 25 °C, c) 45 °C, d) 65 °C, e) 75 °C, with representative AFM images inset.

Importantly, the PDA fluorescence intensity at the endpoint of polymerization (I_max_) also increases an order of magnitude with polymerization temperature, from I_max_(5 °C) ≈20 a.u. to I_max_(65 °C) ≈215 a.u. (**Figure** **3**g). Based on our previous work,^[^ ^9^, ^10^ ^]^ this indicates that a relatively low percentage of the TCDA layer is covalently transferred to the PDMS following polymerization at lower temperatures, even when the monolayers appear to be fully polymerized based on AFM images. Because transfer efficiency to PDMS varies with polymer length,^[^ ^10^ ^]^ the 10‐fold increase in transfer likely results at least in part from increased polymer lengths (degree of polymerization, DP), when polymerization is carried out at higher temperatures. Such an increase would be consistent with increased propagation length within the diradical lifetime.^[^ ^24^ ^]^

To assess the relationship between propagation length and temperature, we measured sPDA lengths in AFM images acquired from samples polymerized at temperatures from 5 to 75 °C (Figure 3). Samples were imaged after polymerization for time periods estimated (from sigmoidal curves in Figure 2) to represent ca. 20% conversion to polymer (e.g., 30 min for 5 °C, 10 min for 45 °C), in order to resolve individual PDAs (Figure 3a–e insets). The number average DP (DP_n_) increased at higher reaction temperatures, up to 65 °C (DP_n,5 °C_ = 97 vs DP_n,65 °C_ = 248). The histogram of PDA lengths for 5 °C also included many polymers with DP < 50, which have a low probability of transfer to PDMS. The histogram of polymer lengths collected for polymerization at 75 °C exhibits a modest decrease in polymer length, in comparison with 65 °C distribution, with DP_n,75 °C_ = 194.

**Figure 4:**
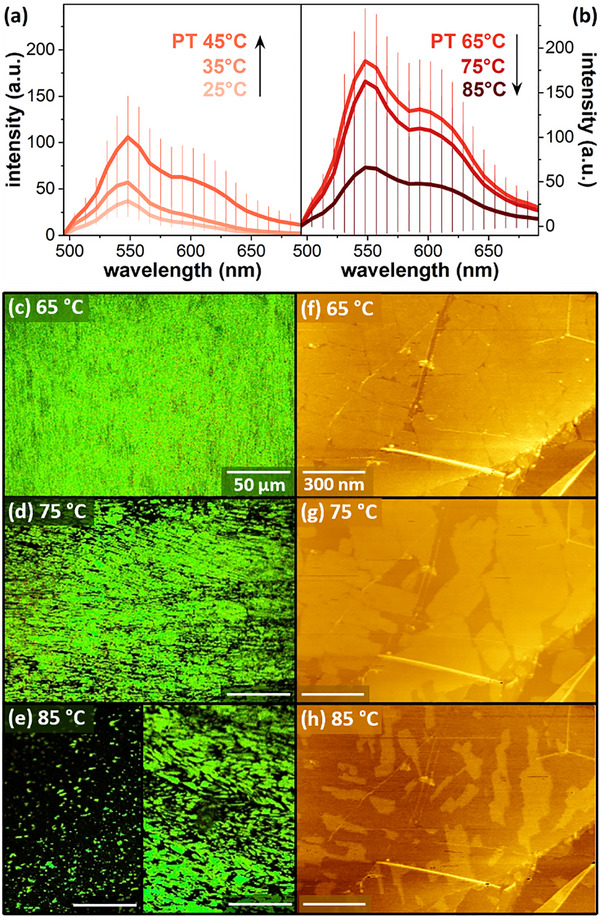
a,b) Spectra illustrating changes in average PDA emission, with (a) increasing emission as polymerization temperatures increases from 25–45 °C, and (b) highest emission for polymerization at 65 °C, decreasing for 75 and 85 °C. c–e) Confocal fluorescence images of PDA monolayers transferred to PDMS at (c) 65 °C, showing high transfer efficiency across the surface, d) 75 °C, illustrating areas of high‐ and low‐efficiency transfer, and e) 85 °C, with two images illustrating increased local and macroscopic variability in transfer. f–h) AFM phase images of TCDA monolayers acquired at elevated imaging temperatures: f) 65 °C, g) 75 °C, and h) 85 °C, illustrating changes in monolayer ordering above 65 °C.

Consistent with single‐polymer AFM measurements, a modest decrease in fluorescence was also observed for samples polymerized at 75 °C (I_max_ = 175 a.u.), and a larger decrease for samples polymerized at 85 °C (I_max_ = 60 a.u.). Confocal fluorescence images (**Figure** **4**c–f) illustrate that the decreases in emission for polymerization at 75 °C and 85 °C derive from increasing microscopic heterogeneity in emission intensity. Because smaller areas of molecular ordering in monolayers are typically less stable, the observed variability may be consistent with disordering of a subset of smaller molecular domains at 75 °C and 85 °C.

To more directly examine the possibility of changes in TCDA monolayer ordering at elevated temperatures, we acquired AFM images of unpolymerized TCDA monolayers at temperatures up to 85 °C. Figure 4 panels (f–h) show a series of images of the same area of a sample acquired as the setpoint temperature was increased from 65 °C to 85 °C; in each case, the temperature was increased at 0.02 °C/s prior to holding at the new setpoint temperature. At 65 °C, the imaged area is predominantly ordered, with small vacancies appearing around domain edges in the image acquired at 75 °C, and larger vacancies at 85 °C.

### 2.4 Models of PDA‒PDMS Transfer Efficiency Versus PDA Length

**Figure 5:**
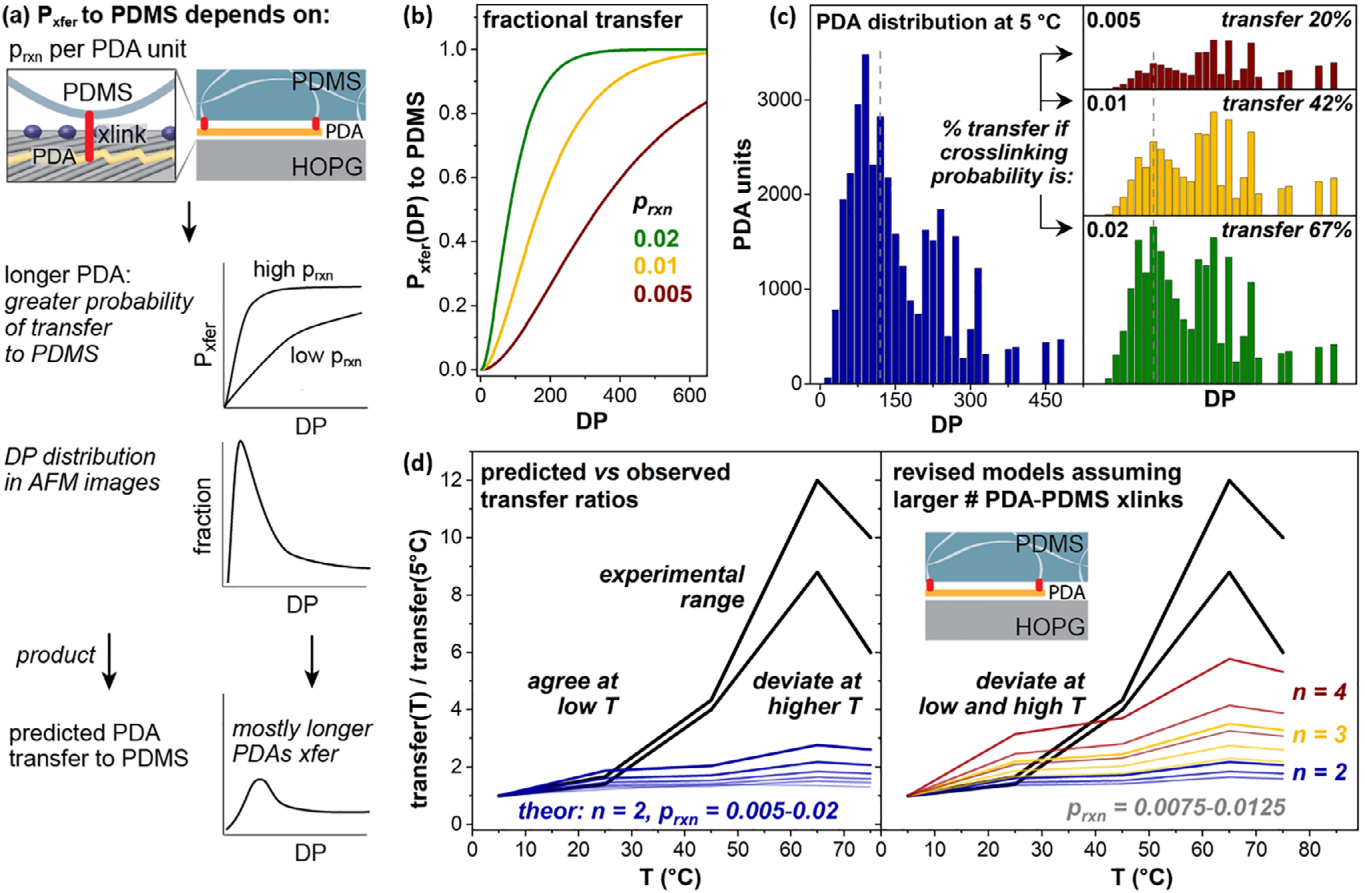
a) Illustration of the relationship between PDA length and probability of successful covalent transfer to PDMS. b) Calculated probability of transfer (P_xfer_) of a polymer of given DP, given a specified probability of crosslinking per PDA unit (p_rxn_). c) Distribution of PDA units contained in polymers of each DP, for polymerization at 5 °C (left), and calculated transfer of the population of PDAs if p_rxn_ = 0.005 (red), 0.01 (gold), or 0.02 (green) with the same vertical scaling as the left panel. d) Predicted versus observed ratios of PDA transfer to PDMS, for monolayers polymerized at temperatures from 5 to 75 °C, using models assuming (left) two covalent linkages required for transfer, and (right) models assuming 2 (blue), 3 (gold) or 4 (red) crosslinks required for transfer.

Our prior work indicates that the efficiency of PDA transfer to PDMS increases with PDA length,^[^ ^9^, ^10^ ^]^ since each PDA repeat unit represents a potential site for crosslinking to the PDMS mesh (**Figure** **5**a, top). Therefore, to understand how much transfer *should* increase with the increased PDA lengths observed for monolayer polymerization at temperatures up to 75 °C, we again used experimentally measured populations of PDA lengths to project the fraction of the monolayer that undergoes transfer, making a series of assumptions regarding the probability of transfer versus DP. The value of p_rxn_ is not straightforward to measure directly, but prior experiments suggest it is in the range of 0.005 to 0.02 for Sylgard 184.^[^ ^10^ ^]^

Figure 5b illustrates the relationship between p_rxn_ and PDA length required for transfer. For instance, if p_rxn_ = 0.005 (red trace), a DP ≈330 is required to have a 50% probability of transferring the PDA (P_xfer_ = 0.5), while if p_rxn_ increases to 0.01 or 0.02 (yellow and green traces, respectively), the DP required for P_xfer_ = 0.5 decreases to ≈170 and ≈85 units, respectively. We then used these probabilities to project the fractional transfer of the populations of polymers measured in Figure 3.

Figure 5c depicts the predicted distribution of PDA units transferred to PDMS. The left panel shows the histogram of experimentally measured polymer lengths after polymerization at 5 °C, with the vertical axis representing the total number of PDA units measured at each polymer length (e.g., 20 polymers comprised of 100 repeat units each would contribute 2000 units of vertical scale). Multiplying the experimentally measured PDA distribution with the predicted fractional transfer curves in Figure 5b, we obtain histograms estimating the number of PDA units that would transfer to PDMS (right panel) given a p_rxn_ of 0.005 (top), 0.01 (center), or 0.02 (bottom). For the population of polymer lengths measured after polymerization at 5 °C, 20%–67% transfer would be predicted, using transfer probability curves that assume 2 or more crosslinks are required per PDA for exfoliation (n = 2).

In Figure 5d, the black lines compare the amount of PDA transfer for PDAs formed at each temperature T with PDAs formed at 5 °C (left panel). Because higher polymerization temperatures led to variable transfer, we calculated ratios using both upper and lower bounds of the ranges of experimentally observed values, leading to the two black lines shown. These are then compared against the theoretically predicted ratios using our previously developed probabilistic models, given a range of p_rxn_ from 0.005 (thickest blue line) to 0.02 (thinnest blue line), and n  =  2 (two or more covalent linkages required for exfoliation). Although the models provide reasonable agreement at lower temperatures, they clearly substantially underpredict the observed difference in transfer for PDAs generated at temperatures 45 °C and higher. While similar models in Figure 5d (right panel) with more stringent requirements for 3 (gold) or 4 (red) crosslinks for exfoliation produce somewhat better agreement at higher temperatures, they then deviate at lower temperatures.

**Figure 6:**
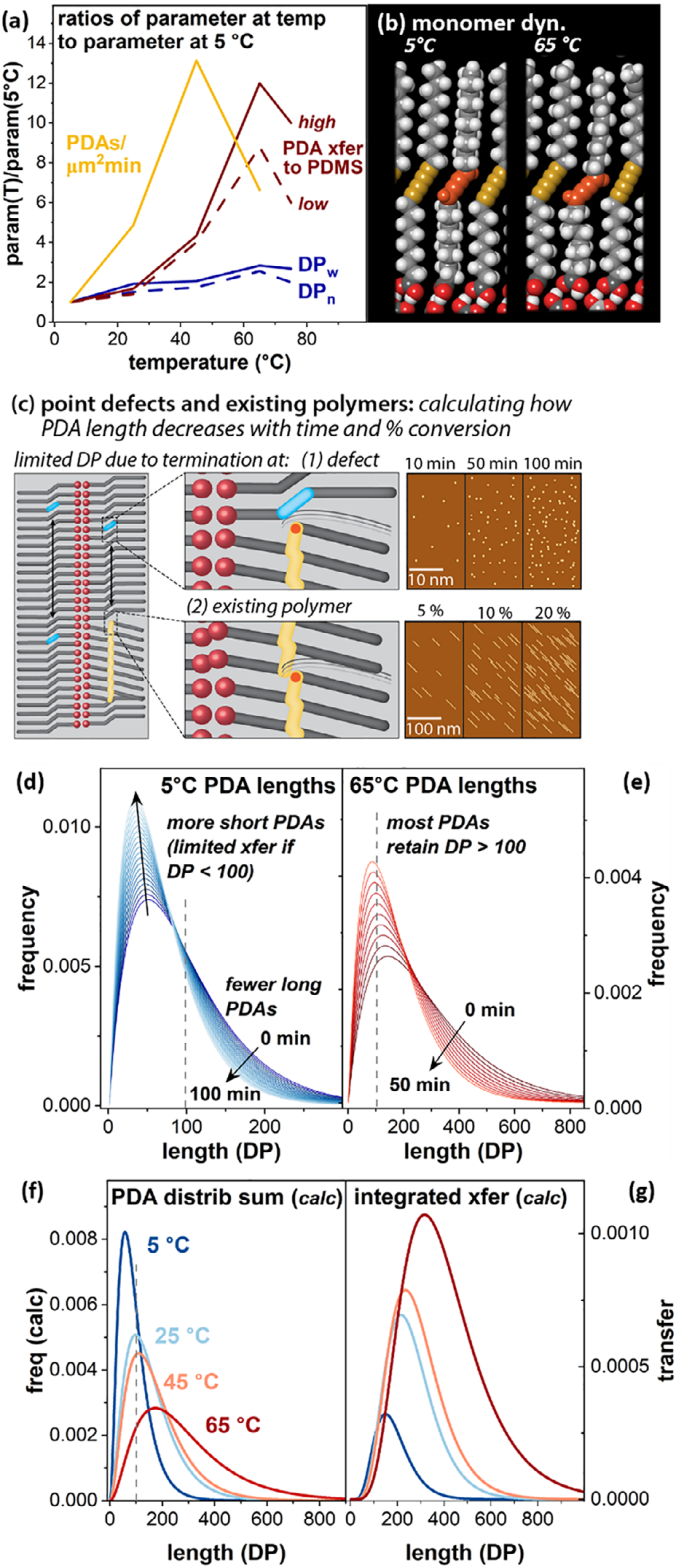
a) Ratios of parameter values at the given temperature to the value at 5 °C for: weight average DP (DP_w_) and DP_n_ (solid and dashed blue traces, respectively), PDA transfer to PDMS as measured by PDA fluorescence emission (red traces, showing typical high and low values across the temperature range, using solid and dashed lines, respectively), and number density of PDAs µm^−^^2^ min^−1^ as calculated from AFM images used to generate histograms in Figure 3 (gold trace). b) Excited radical dimer at 5 and 65 °C. c) Cartoon illustration of the impacts of point defects and existing polymers in decreasing lengths of PDAs formed later in monolayer polymerization. d,e) Calculated modification of 5 and 65 °C PDA length distributions versus time. f) Calculated modified total PDA length distributions incorporating impacts of defects and existing PDAs. g) Calculated transfer to PDMS for length distributions shown in f).

These observations led us to question the implications of another aspect of polymerization evident in the AFM images: higher temperatures result in not only *longer* polymers, but substantially *more* polymers per photon incident on the surface. For instance, histograms for polymerization at 5 °C were collected from AFM images acquired after 30 min UV exposure (Figure 3a inset), whereas histograms for 45 °C and 65 °C were collected after just 10 min UV exposure (Figure 3c,d insets), all at a photon flux of ≈2 photons nm^−2^ s^−1^. Number densities of PDAs were calculated from each image used to generate histograms (**Figure** **6**a, gold trace). Only polymers lying completely within the image area were counted, so the values in Figure 6a represent lower bounds of the actual experimental data. We note that, because PDAs formed at higher temperatures were typically longer, more polymers near image edges were excluded from these data sets, meaning that the observed increase in PDA number densities at higher temperatures may be underestimated. Although local number densities are variable, there is roughly an order of magnitude increase in PDA number density for surfaces polymerized at 45 °C and 65 °C versus 5 °C. However, based on AFM images, polymerization at the lower temperatures appears to produce full conversion to polymer at the timepoints representing the upper segment of the sigmoidal curve, so the higher number density does not by itself explain the large increase in PDA transfer from surfaces polymerized at higher temperatures.

This leads to a further question: how often does photon absorption in the monolayer lead to the generation of a diradical that does *not* form a polymer? The PDA number densities observed at 5 °C after 30 min (1.4 PDA µm^−^^2^ min^−1^) at a photon flux of ca. 2 photons nm^−^^2^ s^−1^ mean that ≈10^8^ photons are incident on the monolayer per polymerization event. While it is not straightforward to measure how the DA absorption cross‐section on the surface changes with temperature, gas‐phase measurements indicate an increase of ≈20% in the absorption cross‐section of butadiyne from 5 to 65 °C,^[^ ^25^ ^]^ a much smaller change in comparison with the observed ≈10‐fold higher PDA number densities observed at 45 and 65 °C. Using models similar to those in Figures 1, we examined conformations of individual butatrienes constructed to mimic the structure of a diradical monomer (Figure 6b). In these models, the alkyl chains, particularly at lower temperatures, typically rotate to adopt an orientation with the backbone zig‐zagging perpendicular to the HOPG (left image). This orients the diradical for possible reactions with species in the environment or with the HOPG. At elevated temperatures, there are increasingly frequent rotational dynamics that would be expected to reorient the radicals relative to the adjacent reacting atoms (right image). Such dynamics may contribute to the observed increase in the number density of polymers observed per unit time at elevated polymerization temperatures (e.g., 65 °C).

Therefore, we suggest that the lower PDA number densities at 5 and 25 °C may be consistent with a scenario in which a large fraction of photoexcitation events do not result in PDA formation, in some cases instead undergoing side reactions that create point defects along the diacetylene rows. Such defects would in turn limit the lengths of polymers formed later in the illumination period (Figure 6c, top), consistent with the lower extents of PDA transfer to PDMS observed experimentally at high monolayer conversion to PDA for low polymerization temperatures. Likewise, existing polymers in the lattice represent additional termination sites (Figure 6c, bottom), meaning that at all polymerization temperatures, it would be reasonable to expect average polymer lengths to decrease throughout the conversion of the monolayer to polymer. However, decreases in DP with conversion should be more substantial at lower polymerization temperatures, since greater numbers of polymers (and thus, greater numbers of termination sites) are formed at a given % conversion.

Although it is challenging to measure individual polymer lengths at high conversion, we carried out calculations of the impacts of the factors described above on the probability of propagation (vs termination) at each reaction step, which can be used to project the distribution of polymer lengths. Figures S15 and S16 (Supporting Information) for more details. Initial probabilities were calculated from the histograms presented in Figure 3, leading to values ranging from p(5 °C) = 0.977 to p(65 °C) = 0.991. We then modified these probabilities with two terms (Figure 6c): one modeled impacts of defects accumulated per unit time due to side reactions upon photoexcitation; one term modeled the impacts of existing polymers in decreasing propagation probabilities as the % conversion of the monolayer to polymer increases. Figure 6d,e illustrate the impacts of these terms on PDA length distributions at 5 and 65 °C, using the experimentally fitted length distribution at the time point at which the histogram in Figure 3 was acquired. These plots depict the impacts if 1 in 10000 monomers is deactivated through non‐polymerization photoreactions per minute, and if existing polymers decrease the propagation probability by 0.015 * fractional conversion.

The impacts of both factors are greatest for polymers formed at 5 °C, which require more time (thus accumulating more photoinduced defects in the monolayer), and generate a greater *number* of pre‐existing polymer defects per unit conversion (since PDAs formed at lower T are shorter, more are required for each percent of the monolayer converted to polymer). Thus, the fraction of polymers with DP < 100 (to the left of the grey dashed line in Figure 6d) increases substantially; this population of polymers exhibits much more limited transfer efficiency to PDMS, contributing to the observed difference in PDA transfer ratio. When the modeled PDA length populations in Figure 6f are subjected to transfer calculations (Figure 6g) similar to those in Figure 5d(right), calculated transfer ratios for I(T)/I(5 °C) are more similar to experimentally observed ratios for 25, 45, and 65 °C *(calc* 3.0, 3.8, 7.4 versus *expt* 1.7, 3.7, and 8.5, respectively). Overall, this reinforces the importance of strategies for improving the efficiency of on‐surface polydiacetylene reactions that are intended for application‐focused work.

## 3 Conclusion

While topochemical reactions rely on molecular ordering to position chemical functional groups for efficient reactions, Ångström‐scale dynamics are also required for the reactions to occur. Here, we have established that striped phase monolayers of TCDA on HOPG exhibit Arrhenius temperature dependence of the reaction kinetics, with an activation energy ≈0.3 eV up to ≈45 °C. The observed value is consistent with a substantial contribution of the flanking alkyl chains to the rate of polymerization, reflecting the relatively strong alkyl‒π interactions that contribute to monolayer ordering. Interestingly, although t_0.5_ does not further decrease above 45 °C, other metrics indicate maximal polymerization efficiency in the range from 45–65 °C, extending well above the bulk melting point of the monomer. Above this range, we observe increasing variability in polymerization metrics, potentially consistent with partial disordering of the monolayer at high temperature.

Greater polymerization efficiency at higher temperature results from both greater propagation lengths (≈2.5× increase from 5 °C to 65 °C), but also higher number densities of polymers formed from a given number of incident photons. This is an important consideration, since reaction cross‐sections are substantially lower for the on‐surface DA polymerization than for equivalent bulk polymerization, with ≈10^8^ photons producing one polymerization event at room temperature in the system used here. TCDA molecular sheets formed at 65 °C are much more robust than those formed at lower temperatures, exhibiting ≈10× greater efficiency of covalent transfer to PDMS, in comparison with polymerization at 5 °C. Although it becomes challenging to resolve individual polymers as the reaction approaches completion, we suggest that this difference may arise in part due to excitation events at lower temperatures that do not result in polymer formation and instead result in other reactions, producing defects in the DA array. These defects in turn, would be expected to act as termination sites for subsequent polymerization events, decreasing the DP values of PDAs formed later in the reaction process.

More broadly, we suggest that thermally assisted photopolymerization has the potential to provide significant advantages in generating efficiently linked molecular networks for nanostructured chemical interfaces, both by increasing propagation rates and by increasing rates of initiation/dimer formation, thereby limiting photodamage. While here we have characterized the temperature‐dependence of polymerization in relation to TCDA, a widely available commercial alkyldiacetylene monomer, the guiding principle of carrying out the on‐surface photopolymerization at a temperature just below the monolayer disordering temperature is likely to maximize efficiency for other diacetylene monomers, pointing to the importance of characterizing such order–disorder transition temperatures more routinely.

## Conflict of Interest

The authors declare no conflict of interest.

## Supporting information



Supporting Information

## Data Availability

The data that support the findings of this study are available in the supplementary material of this article.
