# Supplementary material for: Topochemical Photopolymerization above the Bulk Melting Temperature in Noncovalent Monolayers on Graphite
Source: Small. 2025 Sep 18;21(45):e09179. doi: 10.1002/smll.202509179 (PMC12614152; doi:10.1002/smll.202509179)
Supplement: Supplementary file 1 — Supporting Information [file SMLL-21-e09179-s001.docx]

Supporting Information

**Topochemical Photopolymerization Above the Bulk Melting Temperature in Noncovalent Monolayers on Graphite**

Joseph A. Garfield†, Tobiloba Awoyemi†, Emmanuel K. Nava†, Shelley A. Claridge*,†,‡

†Department of Chemistry, Purdue University, West Lafayette, Indiana 47907

‡Weldon School of Biomedical Engineering, Purdue University, West Lafayette, Indiana 47907

*Address correspondence to: claridge@purdue.edu, (phone) 765-494-6070

**Materials.**10,12-Tricosadiynoic acid (≥98 %) and chloroform (contains 100-200 ppm amylenes as stabilizer, ≥99.5%) were purchased from Millipore Sigma (St. Louis, MO) and used as received. Sylgard 184 (DOW manufacturer) elastomer kits, containing flowable silicone elastomer base and crosslinker, were purchased from Newark (Richfield, OH). AFM probes, Bruker RFESP-75 (0.01–0.025 Ω · cm Antimony (n)-doped Si, nominal force constant 3 N/m and radius of curvature <12 nm) were purchased from Bruker AFM Probes (Camarillo, CA). Highly oriented pyrolytic graphite (HOPG) substrates, grade ZYB, were purchased from SPI Supplies (West Chester, PA). 25-mm PTFE syringe filters were acquired from VWR (Radnor, PA) and used to filter TCDA amphiphiles. Control Cure UV filter sleeve 4’ amber for T4 fluorescent bulbs purchased from UV Process Supply Inc and used to filter laboratory lighting. Milli-Q water (≥18.2 MΩ ∙ cm resistivity) was used in all experiments where water was required. Peltier plate assembly equipment including the cold plate cooler (CP-031HT, 12 V-DC), moisture resistant thermistor with 900 mm long 26 awg wire leads (MP-3193) and thermometric temperature controller (TC-48-20) were purchased from TE Technology Incorporation (Traverse City, MI). Power supply for cold plate cooler (LRS-150F-12, AC-DC converter 1 output 12 V, 85-264 VAC, 120-370 VDC input), and toggle switch DPDT panel mount (2057-SW-T1-4B-B-K2A-ND, used to transition from hot to cold mode), were purchased from DigiKey (Thief River Falls, MN). Handheld UV lamp (UVP 95-0201-01 Model UVLS-28 EL Series 2UV, 8 Watt, 254 nm shortwave/365 nm longwave, 14.8-inch length, 115 V) was purchased from Capitol Scientific (Austin, TX). Ultraviolet photon flux was recorded using a TR-74UI-S High Precision Illuminance, UV, Temperature and Humidity Data logger. Relative humidity was measured using an Onset HOBO MX1101 Bluetooth Temperature and Humidity Data Logger. Adhesive Tape was purchased from DigiKey (P/N: 1067-9472LE-0.5-60-ND). Self-adhesive polyimide fast response surface thermocouples (SA1XL) were purchased from DwyerOmega (Michigan City, IN). A DwyerOmega Multilogger thermometer (model HH506RA) was used for surface temperature readings.

**Peltier plate assembly design and assembly.** A Peltier plate apparatus was designed and fabricated, enabling DA polymerization on HOPG to be carried out at desired setpoint temperatures, in an inert environment. Sample setpoint temperature was maintained using a Peltier cold plate cooler (CP-031HT, TE Technologies Inc., Traverse City, MI), providing efficient direct cooling or heating from 0–100 °C. The setpoint is input using a thermometric temperature controller (TC-48-20, TE Technologies Inc., Traverse City, MI); a thermistor continuously monitors the temperature at the sample plate. Four 3D printed custom support members maintain the position of the Peltier plate above the optical breadboard, for efficient thermal dissipation from the fan to surroundings. A UV lamp (UVP-95-0201-01, Capitol Scientific, Austin, TX) is independently positioned at a fixed height above the Peltier plate, using additional custom 3D-printed supports. To achieve a photon flux equivalent to that used in our previous work (~6900 photons nm^-2^ h^-1^ , or ~2 photons nm^-2^ s^-1^), the UV lamp was mounted *ca*. 2 cm above the surface of the cold plate. Photon flux was quantified using a TR-74UI-S UV data logger (MicroDAQ, Concord, NH). The apparatus was housed in an environmental chamber (818-GB, Plas-Labs, Lansing, MI), with an N_2_ feed used to control the gas-phase composition during polymerization, and an Onset HOBO MX1101 Bluetooth Temperature and Humidity Data Logger to monitor humidity levels.

**Langmuir-Schaefer transfer to generate striped diacetylene films.** Striped phase diacetylene (DA) films were prepared based on a Langmuir-Schaefer conversion method we have reported previously^1-3^ with minor modifications, described briefly here. Langmuir-Schaefer transfers were performed using a microTrough XL Langmuir-Blodgett trough (Kibron Inc., Helsinki, Finland) with a customized temperature-controlled transfer stage. HOPG substrates were cleaved immediately prior to sample deposition. Substrate setpoint temperature was maintained at 35 °C. This temperature is somewhat lower than we have typically used to maximize domain size, but high enough to avoid subphase condensation on the HOPG as it approaches the Langmuir film, and low enough to avoid thermal DA polymerization. For the LS transfer, 34.5 µL of 0.75 mg/mL of the desired amphiphile in CHCl_3_ was deposited on a subphase of deionized water (~ 18 MΩ · cm) as 1-µL droplets distributed across the trough surface. The system was allowed to equilibrate for 15 min prior to compression, which was carried out by sweeping the moveable barriers inward at 2.55 mm/min until the target mean molecular area (typically 30 Å^2^/chain, corresponding to a surface pressure of ~4‒8 mN/m) was achieved. Freshly cleaved HOPG substrates were mounted on the transfer stage, oriented nearly parallel to the air–water interface, and brought into contact with the subphase at a rate of 2 mm/min using the automated dipper. Following a 1-min period in contact with the subphase, the substrates were then lifted out of contact at the same rate, then blown dry with UHP N_2_. DA monolayers were polymerized for the time stated in the manuscript (0 – 100 min) by placing them on the Peltier plate at the desired set-point temperature for 30 min before polymerizing under a UV lamp (λ_max_ = 254 nm, 8 W), with ~2 cm between the lamp and the substrate. To ensure equivalent photon flux, this parameter was measured at several locations under the lamp, and samples were placed in locations with equivalent flux.

**Measuring temperature dependence of the kinetics of striped phase DA monolayer polymerization.** To elucidate the Arrhenius temperature dependence of polymerization kinetics of striped phase DA polymerization, we quantified the rate of conversion to PDA, at photopolymerization temperatures from 5–65 °C. All photopolymerization reactions were performed in an environmental chamber flooded with UHP N_2_ until relative humidity levels reached 10 % or lower, to ensure an inert environment. Utilizing the Peltier plate assembly described above, substrates were positioned on the plate at locations selected to ensure uniform UV intensity Samples were equilibrated at the set-point temperature for 30 min, then maintained at the same temperature and irradiated with a UV lamp mounted ~2 cm above the surface of the Peltier plate. After the desired polymerization time, samples were removed from the UV-illuminated area and stored at room temperature under UV-free conditions prior to AFM imaging or transfer to PDMS.

**Covalent transfer of sPDA layers from HOPG to PDMS.** Transfer of sPDA layers from HOPG to PDMS was carried out using a minor adaption of our previously reported protocol.^4^ Sylgard 184 silicone base and curing (crosslinking) agent were mixed in a 10:1 (*mass/mass*) ratio. After components were thoroughly mixed with a stir bar at 200 rpm (~10 min), the mixture was poured over HOPG substrates functionalized with sPDA monolayers. The PDMS-coated substrates were placed in a vacuum chamber for at least 30 min, or until no bubbles were observed. PDMS-coated substrates were subsequently cured at a relatively low temperature (35 °C) for 24 h to avoid thermal polymerization of PDAs (see further discussion of condition optimization, in a later section of the Supporting Information). The cured PDMS was then gently exfoliated from the HOPG and stored under ambient conditions prior to further characterization.

**Atomic force microscopy (AFM) imaging.** AFM images were acquired under an ambient environment in tapping mode, using either a Veeco MultiMode with a Nanoscope V controller or a Veeco Dimension 3100 AFM with a Nanoscope IIIa (Bruker Instruments, Billerica, MA). Imaging was carried out using Bruker RFESP-75 tips (nominal force constant 3 N/m and radius of curvature <12 nm).

**Temperature-controlled atomic force microscopy (AFM) imaging.** AFM images were acquired using a Cypher ES AFM (Oxford Instruments, Santa Barbara, CA). The setpoint of the temperature-controlled stage was varied from 25 °C – 85 °C, using AC Air Topography mode, with images acquired at 10 °C increments. A 0.02 °C/s temperature ramp was used when the setpoint was changed, to avoid AFM tip collisions with the surface due to thermal drift. Cantilever oscillation and calibration were carried out utilizing blueDrive photothermal excitation to maintain stable, high-resolution images. Phase shift values of 90° and above were maintained (attractive interaction regime), to minimize monolayer restructuring due to surface contact. Imaging was carried out using Bruker RFESP-75 tips (nominal force constant 3 N/m and radius of curvature <12 nm).

**Scanning electron microscopy (SEM) imaging**. SEM imaging of monolayers on HOPG under high magnification was performed using a Teneo VS SEM (FEI Company, Hillsboro Oregon) at a working distance of 5–7 mm using the segmented in-lens T3 secondary electron (SE) detector. For image acquisition, an accelerating voltage of 5 kV, beam current of 0.4 nA, and 32-µm diameter aperture were typically utilized. Samples were attached to the SEM specimen stage with conductive carbon tape. All images were collected with a 1536 × 1024 pixels resolution at a 30-µs dwell time, unless otherwise specified.

**Confocal fluorescence microscopy and spectral imaging.** Fluorescence images and emission spectra were acquired using a Zeiss LSM 880 Axio Examiner upright confocal microscope. Diynoic acid-functionalized PDMS samples were imaged with a 20× objective (plan-apochromatic, dry, NA = 0.80, no coverslip) immersed in water. Image excitation was carried out using a 488-nm Ar laser at 100% power. Emitted fluorescence was detected by a 32-channel GaAsP spectral photomultiplier detector with a pinhole size set to 1 Airy unit. All fluorescence images and corresponding spectra were collected at a resolution of 2856 × 718 pixels with 8-bit depth. Unidirectional horizontal scans were averaged 4 times/line with a dwell time of 3.77 μs/pixel. Emission spectra were collected from 495–691 nm with a bin width of 8.9 nm when evaluating the PDA transfer.

**AFM and SEM image analysis.** AFM and SEM images were processed with Gwyddion SPM software (<http://gwyddion.net>) and ImageJ (https://imagej.net/ij/download.html). Prior to quantitative analysis, mean plane subtraction and row alignment procedures, such as fitting to median or median differences, were performed for all raw data files in Gwyddion. Gwyddion software was also used to perform polymer counting and length measurements on polymerized samples. ImageJ was additionally used to perform polymer counting in images containing contiguous polymerized blocks.

**Analysis of AFM images to quantify populations of polymer lengths on HOPG**

To assess the extent of conversion from monomer to polymer under temperature-controlled polymerization conditions, we quantified features in AFM images as described below, comparing images acquired from samples polymerized at set-point temperatures from 5–75 °C , at approximately 20% conversion. Polymerization times utilized to achieve ~20% conversion were: 30 min for 5 °C, 20 min for 25 °C, and 10 min for 45, 65, and 75 °C.

Polymerized diacetylenes (PDA) were identified based on topographically protruding features as described previously.^8^ For each PDA feature identified, we used the measured feature length to calculate the degree of polymerization (DP) as:

$$DP=\frac{polymer length}{0.50 nm}$$

Due to the cooperativity of diacetylene polymerization, images in some cases contained blocks of adjacent polymerized molecular rows, in which individual PDAs can be difficult to identify individually. In such cases, we determined the number of PDAs by dividing the width of the protruding block by the molecular length of the amphiphile:

$$\# PDA= \frac{block width}{3 nm}$$

**Measurement of degree of polymerization.** To assess differences in DA polymerization based on temperature, we calculated both number-average and weight-average degrees of polymerization (DP_n_ and DP_w_); DP_n_ values are shown in Figure 4 of the main manuscript. In bulk polymer experiments, these values would typically be calculated from the number-average and weight-average molecular weights (M_n_ and M_w_, respectively), using the following formulas:

$$\mathrm{DP}_{n}=\frac{M_{n}}{M_{0}}$$

$$\mathrm{DP}_{w}=\frac{M_{w}}{M_{0}}$$

where M_0_ represents the molecular mass of the DA monomers.

Because the AFM measurement strategy measures polymer lengths rather than polymer mass, the DP of each polymer is recorded directly using the formula given on the previous page, and these values are used to calculate DP_n_ and DP_w_ as follows, where N_i_ is the number of polymers with degree of polymerization DP_i_ :

$$\mathrm{DP}_{n}=\frac{\sum_{i} N_{i}\mathrm{DP}_{i}}{\sum_{i} N_{i}}$$

$$\mathrm{DP}_{w}=\frac{\sum_{i} N_{i}\left( {DP}_{i} \right)^{2}}{\sum_{i} N_{i}\mathrm{DP}_{i}}$$

**Analysis of fluorescence images.** Fluorescence images for timepoint studies were analyzed and converted to .bmp format *via* Zeiss Zen software (Blue version) (Carl Zeiss NTS Ltd., Jena, Germany). Fluorescence intensities of transferred TCDA monolayers were acquired from 3 samples at each selected polymerization time point, with times ranging from 0 – 100 minutes.

For each image, fluorescence emission was quantified in a region of interest (ROI) selected from within the in-focus area, typically with one dimension of the ROI similar to the image edge length. Fluorescence emission was measured in three different areas of each sample, to calculate means and standard deviations of emission intensities. If samples included focused regions with obvious differences in brightness, we included ROI analyses on areas with varying brightness to capture sample variability.

**Large AFM images of unpolymerized TCDA on HOPG.** Figure 4ab in the main manuscript utilizes a small-scale AFM image to demonstrate ordered molecular domain structures for TCDA assembled in striped phases on HOPG. Figure S1 provides larger AFM images showing domain structures of unpolymerized (Figure S1a) and polymerized (Figure S1b) TCDA, to facilitate visual inspection.

**
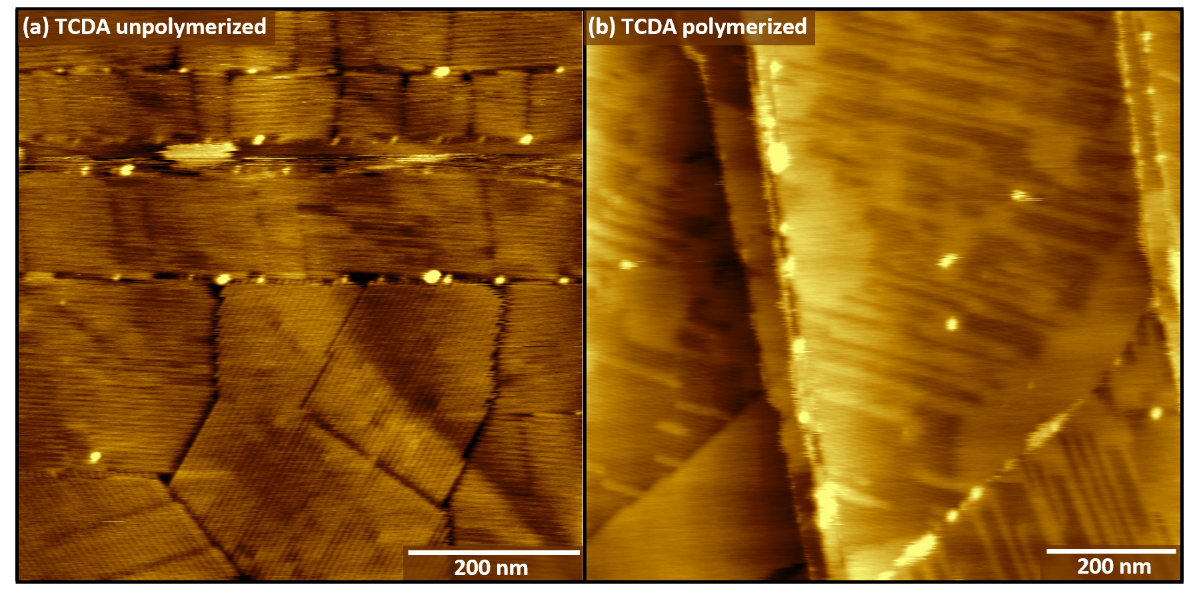
**

**Figure S1.** AFM image illustrating typical domain structures of TCDA, both (a) unpolymerized and (b) polymerized at 45°C.

**Larger SEM images of TCDA.**  In the main manuscript, Figure 3c shows an SEM image of TCDA, illustrating the morphology of monolayers over microscopic scales. Here, we present a larger SEM image of TCDA (Figure S2). Here we have selected a surface with small triangular unfunctionalized regions indicated by the dark contrast; brighter contrast regions are TCDA monolayers.

*
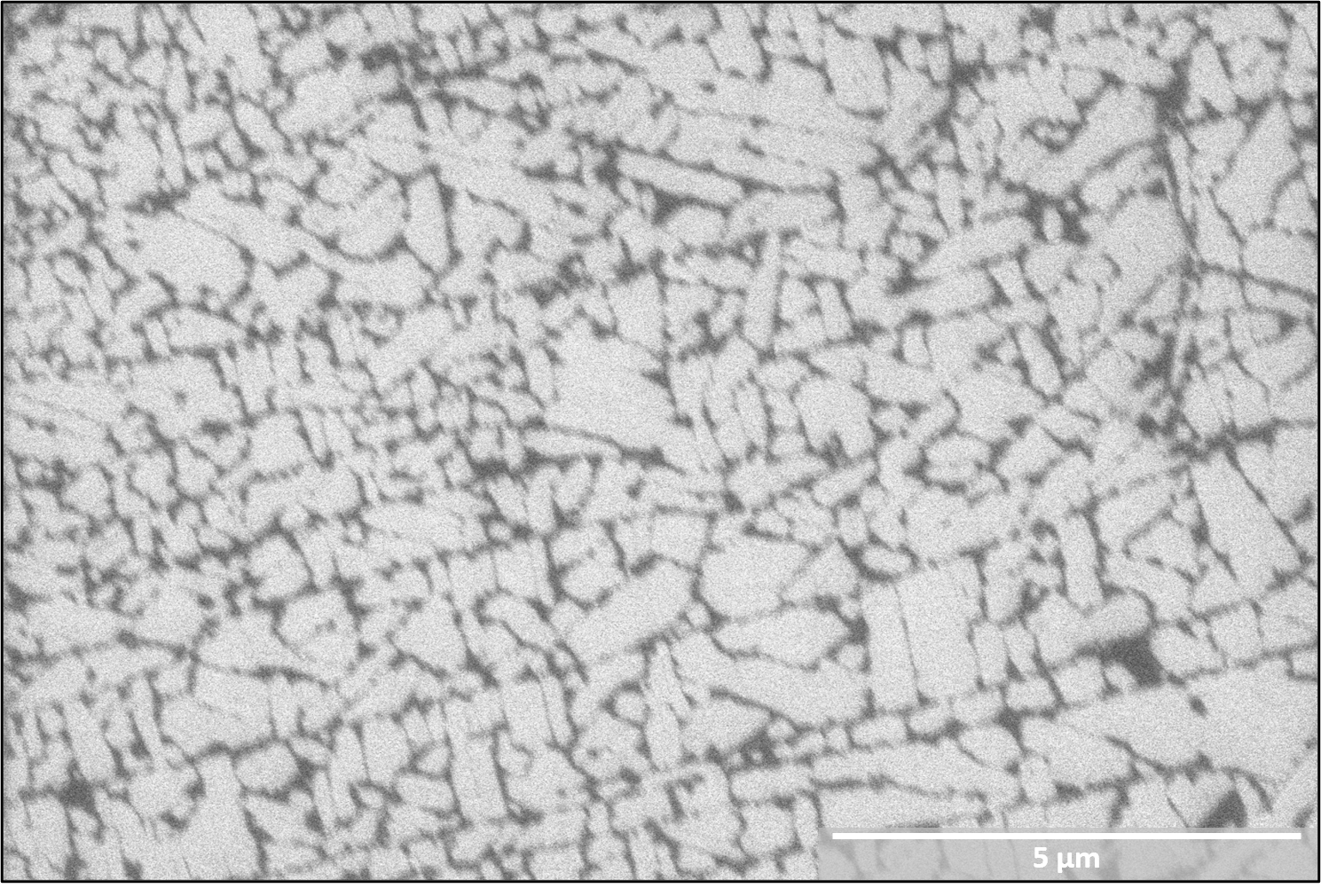
*

**Figure S2.** SEM image of TCDA assembled on HOPG.

**Representative confocal images of TCDA polymerized for 0–60 min at 5–45 °C.** Figure 3 of the main manuscript illustrates kinetics for the conversion of TCDA monomer to polymer at polymerization temperatures from 5–45 °C. The extent of polymerization is quantified based on observed PDA fluorescence emission intensity after transfer of polymerized TCDA monolayers to PDMS. Here, we include a series of representative images of TCDA/PDMS to facilitate visual inspection. Fluorescence emission intensity was quantified based on at least 3 different microscopic regions of interest (ROIs) identified on each of at least 3 different samples, for a minimum of 9 regions analyzed per condition, as described in the analysis section of the Supporting Information.

*
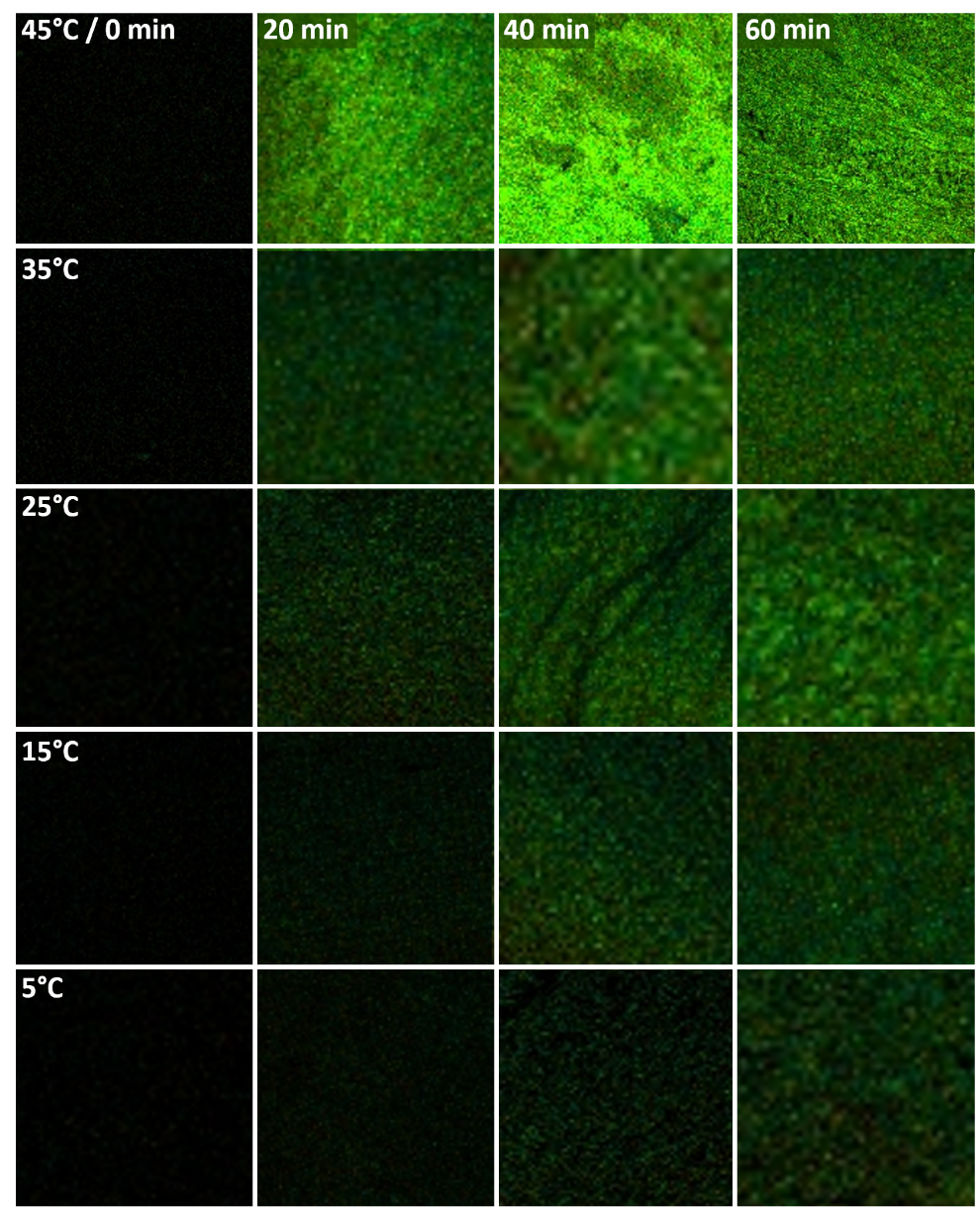
*

**Figure S3.** Confocal images of TCDA/PDMS samples prepared from monolayers polymerized at set-point temperatures from 45 °C (top row) to 5 °C (bottom row), and polymerization times from 0 min (left column) to 60 min (right column)

**Larger AFM images of TCDA polymerized at 5 °C, 25 °C, 45 °C, 65 °C, and 75°C.** In the main manuscript (Figure 4), small-scale AFM images are used to demonstrate the increase in average PDA length associated with polymerization at higher temperatures. Here, we show larger scale AFM images to assist in visual inspection. Note that for the highest temperatures, images 400 nm scale bars are used to show complete polymers within the image frame.


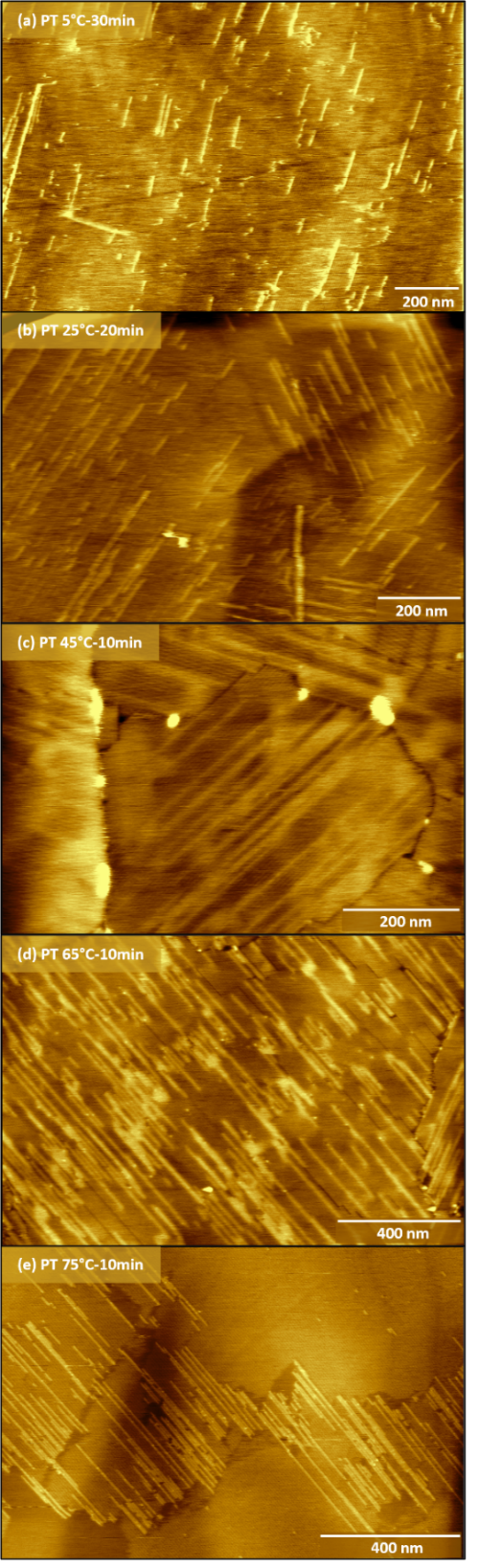


**Figure S4.** Large-scale AFM images of TCDA monolayers polymerized on HOPG at (a) 5 °C for 30 min, (b) 25 °C for 20 min, (c) 45 °C for 10 min, (d) 65 °C for 10 min, and (e) 75 °C for 10 min.

**Impacts of monolayer assembly temperature on polymerization.** Because some DAs undergo thermal polymerization at elevated temperatures (in some cases as low as 60 °C), we carried out experiments aimed at establishing t_1/2_ (t_0.5_) using monolayers assembled at relatively low temperatures (30 °C). However, in previous work, we have also observed that for many molecules, assembly at elevated temperatures produces longer-range molecular ordering.^5-6^ Larger average molecular domain sizes would have the potential to increase average PDA lengths, in turn increasing fraction of the monolayer that can be transferred to PDMS. Therefore, we also carried out experiments in which monolayers were assembled at moderately elevated temperatures (55 °C). For polymerization at 25 °C (Figure S5a) and 35 °C (Figure S5b), we found that this led to similar fitted values of t_1/2_ (solid lines) in comparison with experiments presented in the main manuscript based on monolayers assembled at 30 °C (dotted lines in Figure S5a,b). Thus, we suggest that molecular domain size is not substantially changing the value of E_a_ calculated in the main manuscript.

**
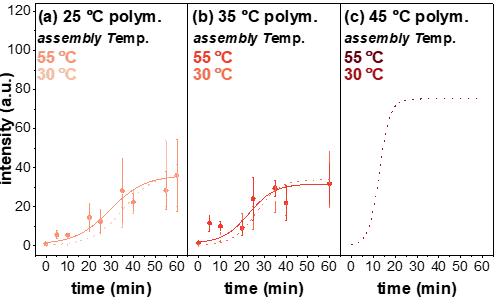
**

**Figure S5.** Comparison of polymerization as measured based on fluorescence emission after transfer to PDMS, for monolayers assembled at 55 °C (solid trace) *vs.* 30 °C (dashed trace) and polymerized at (a) 25 °C or (b) 35 °C.

**Averaged fluorescence spectra 5–45 °C.** In the main manuscript, we show average fluorescence spectra for monolayers assembled at 35 °C and subsequently polymerized at a setpoint temperature of 25 °C. Below, we additionally show spectra for samples polymerized at setpoint temperatures from 5–45 °C.

**Figure S6.** Confocal spectra for monolayers polymerized at (a) 5 °C, (b) 15 °C, (c) 35 °C, and (d) 45 °C, at timepoints ranging from 0 min (blue) to 100 min (yellow).

**Average fluorescence spectra for unpolymerized samples 5–85 °C.** Because we tested photopolymerization efficiency at temperatures up to 85 °C, we carried out control experiments evaluating the extent of thermal polymerization in the absence of UV illumination. Unpolymerized TCDA monolayers were placed on the Peltier plate at controlled temperatures (CT) for 30 min (5–45 °C) or 80 min (65–85 °C) before being transferred to PDMS and then imaged (Figure S7). Heating alone produced minimal PDA fluorescence (0.5 a.u. for 5‒45 °C, 1.5 to 3.5 a.u. for 65‒85 °C) in comparison with the same temperature under UV illumination, as shown in the main manuscript.

**Figure S7.** Confocal spectra for unpolymerized monolayers maintained at elevated temperature for 30 min at 5–45 °C, or 80 min at 65–85 °C before transfer to PDMS.

**Optimizing PDMS curing temperature: RT *vs.* 35°C.** In order to maximize the utility of PDA fluorescence on PDMS as a readout of extent of polymerization, we assessed multiple PDMS curing conditions, in order to find conditions that minimized curing time while also minimizing observed PDA fluorescence from ‘0 min’ samples not exposed to UV illumination. Although PDMS curing can be achieved at room temperature (RT), a 39 h curing cycle is necessary to ensure complete cross-linking. In addition, room temperature curing can decrease PDMS modulus, complicating exfoliation. Curing for 24 h at 35 °C (Figure S8) produced equivalent fluorescence emission for polymerized samples, and <1 a.u. of emission at the PDA emission maximum (λ_em_ = 548 nm) from unpolymerized samples (visible in the truncated graph showing the region near the baseline, in Figure S8b). Thus 35 °C was selected as the PDMS curing temperature for experiments in the main manuscript.

**Figure S8.** Confocal spectra for unpolymerized (0 min) and polymerized (60 min) TCDA monolayers after curing in contact with PDMS for 24 h or 48 h. (a) Full-scale spectra. Panel (b) shows the same spectra as panel (a), in the region from 0 to 5 a.u., in order to clearly show the minimal fluorescence emission from unpolymerized samples.

**Evaluating polymerization kinetics at 85 °C.** As described in the manuscript, we carried out photopolymerization at setpoint temperatures up to 65 °C (t_1/2_ = 24.6 min) and 85 °C (t_1/2_ = 23.0 min). These experiments showed an increase in t_1/2_ with respect to 45°C (t_1/2_ = 12.8 min). However, fits for 85°C exhibited large deviations (typically ±130 a.u.) across the polymerization timepoints. Here we present sigmoidal fits of time-dependent conversion of TCDA to sPDA for 5°C–45°C (left panel) and 65°C–85°C (right panel) for clearer visual comparison between the temperature conditions.

**Figure S9.** Sigmoidal fits of time-dependent conversion of TCDA to sPDA based on λ_em_ intensities @548nm for 5°C–45°C (left panel), 65°C–85°C (right panel).

**Determining rate constant for surface diacetylene polymerization**. Solid-state polymerization of diacetylene crystals has been studied extensively, with polymer conversion induced thermally, photochemically, or by gamma radiation.^7-18^ The conversion to polymer over time has been monitored with a wide variety of methods including extraction of monomer,^7^ absorption spectroscopy,^15^ differential scanning calorimetry,^9,11^ Raman spectroscopy,^10^ and electron spin resonance spectroscopy.^13-14^ Overall, the reaction is typically characterized by an induction period followed by an autocatalytic acceleration towards full conversion to polymer. While the interplay of structural influences on polymerization rate is somewhat complex, key aspects of system behavior are typically described in a simplified manner by treating each reaction regime as though it exhibits first-order kinetics.^7-16^ As described by Sworakowski and coworkers:^12^ “The increase of the polymer content is indeed, to a good approximation, described by first-order kinetics”; as further described by Baughman:^8^ “[the reaction is] in general not first order in any fundamental sense. However, comparison with experimental results is expedited by representing calculated curves by an induction period and a first order rate constant K, corresponding to the slope of -ln(1-X) versus t between X=0.2 and 0.7.”

Throughout the body of work describing solid-state diacetylene polymerization, metrics describing the relationship between temperature and the chemical kinetics have varied, but share significant similarities.^7,9,13,16^ Quantities measured to calculate activation energy have included: inverse of time required to reach to 50% polymerization (t_0.5_ or t_1/2_), or time from 10 to 50% polymerization,^7^ time to maximum polymerization (autocatalytic regime) and time to maximum polymerization rate (typically at t_0.5_);^9^ alternatively, rate constant has been calculated based on a semilogarithmic plot in the autocatalytic regime.^8,10-12,15^ STM-based characterization has also utilized number density of polymers to calculate rate constant.^16^

In the work described in the main manuscript, we bridge molecular-scale and microscale characterization of surface polymerization using a method we have developed previously,^17^ in which diacetylene monolayers are photopolymerized on HOPG, and the extent of polymerization is assessed based on the amount of PDA that is transferred to PDMS cured in contact with the monolayer. This method, while indirect, provides a means to assess the extent of polymerization over larger areas of the monolayer than can be easily measured by AFM or other scanning probe methods. Previously, we have found that the extent of transfer vs polymerization time exhibits sigmoidal characteristics expected of diacetylene polymerization.^17-18^

In the work carried out herein, photopolymerizing monolayers at elevated temperatures resulted in greater average polymer lengths, higher number densities of polymers, and thus more rapid overall conversion to polymer. As expected, the reaction was characterized by a slow induction period, followed by a period of rapid polymerization (autocatalytic regime). In line with the body of prior work described above,^7-16^ the DA polymerization was analyzed within the framework of first-order reaction kinetics. Since a range of metrics has been used previously to calculate rate constants for similar reactions in 3D crystals, we compared values of k calculated using the following methods: calculating rate constant k from t_1/2_ values extracted from sigmoidal curve fits of polymerizations carried out at setpoint temperatures from 5–65°C (k_ca_), semilogarithmic plot of conversion based on experimental data in the autocatalytic regime (k_ex_), and a semilogarithmic plot of conversion based on values of the fitted sigmoidal curve in the autocatalytic regime (k_s_).

Assuming first-order kinetics, rate constant k can be calculated based on the value of t_1/2_, utilizing the following equation:

$$t_{1/2}=\frac{0.693}{k_{ca}}$$

Table S1 below shows the results from such calculations carried out using t_1/2_ values from sigmoidal curves fitted to experimental data describing conversion to polymer at reaction temperatures from 5 to 65 °C.

**Table S1**. Calculated rate constant k for the respective photopolymerization temperatures using t_1/2_ determined from sigmoidal curves fitted to experimental data collected from samples polymerized at setpoint temperatures of 5–65°C, as shown in the main manuscript.

As described in the main manuscript, the amount of PDA transferred to PDMS is quantified based on fluorescence emission at the PDA emission maximum wavelength (*I_value_)*; previously, we have demonstrated that these values are correlated with the extent of on-surface polymerization on HOPG, as well as with average PDA length (since shorter PDAs are less likely to transfer). Thus, the fractional conversion of monomer to PDA at a given timepoint is approximated as a ratio with the maximum fluorescence intensity at the endpoint of polymerization (*I_max_*), which is established from the sigmoidal fit:

$$X=fraction of monomer converted to polymer= \frac{I_{value}}{I_{max}}$$

This allows experimental data to be plotted using equations for first-order reaction kinetics, enabling estimation of rate constants for each photopolymerization temperature (Figure S10a-j). A similar strategy was implemented by Alekseev and coworkers for quantifying PDA polymerization kinetics in 3D crystals.^15^

$$\ln\left( 1-X \right)=-k_{y}t$$

$$k_{y}=k_{ex} or k_{s}$$

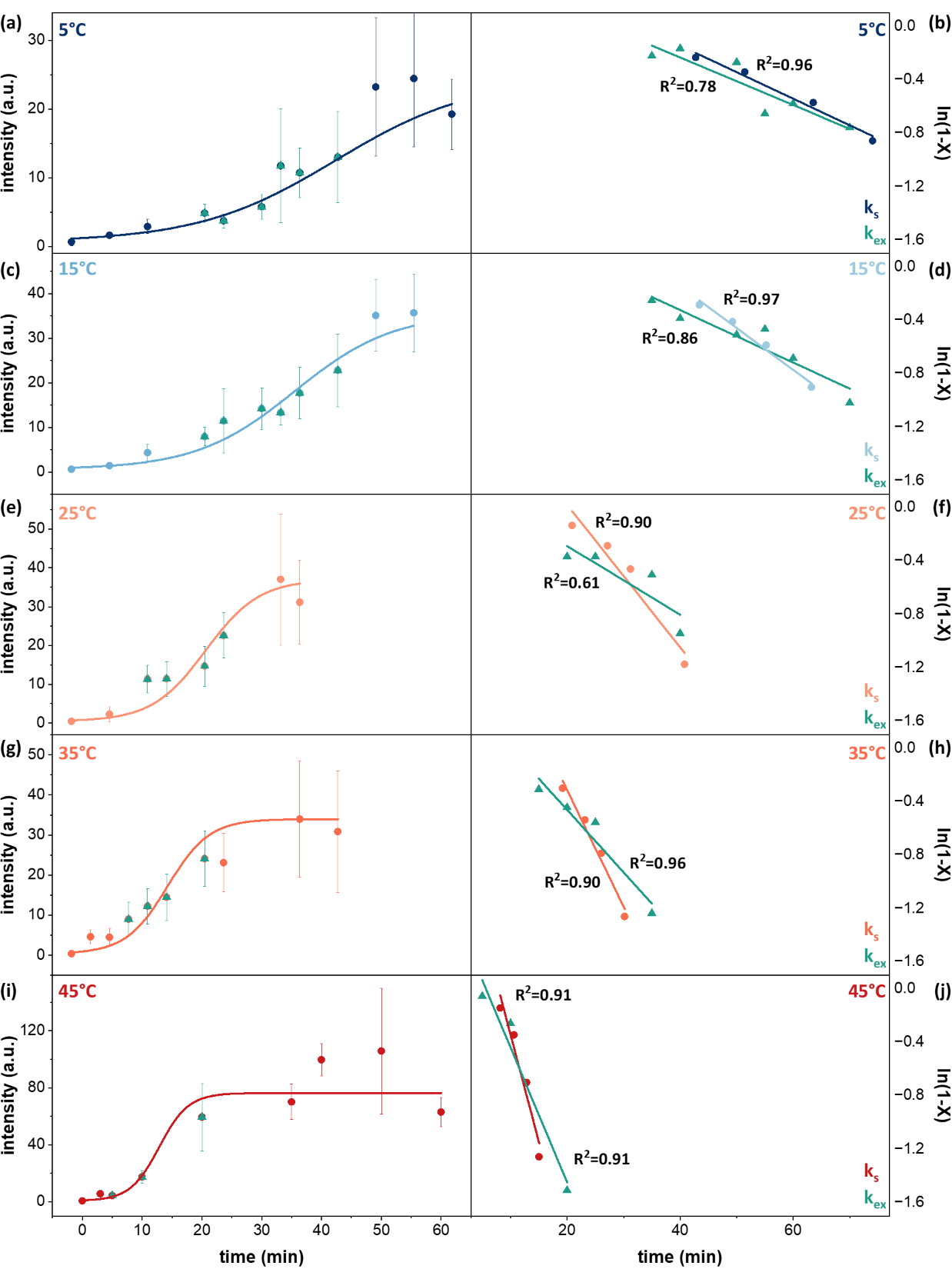


**Figure S10.** (a,c,e,g,i) Sigmoidal fits of time-dependent conversion of TCDA to sPDA based on fluorescence emission intensities at λ_em_ = 548 nm, for polymerization carried out at: (a) 5 °C, (c) 15 °C, (e) 25 °C, (g) 35 °C, or (i) 45 °C. (b,d,f,h,j) Semilogarithmic plots of conversion versus time for the same temperature conditions, respectively. Green markers in each sigmoidal fit graph indicate data points used in semilogarithmic graph to generate fits used to determine k_ex_. The other linear fit determines k_s_.

**
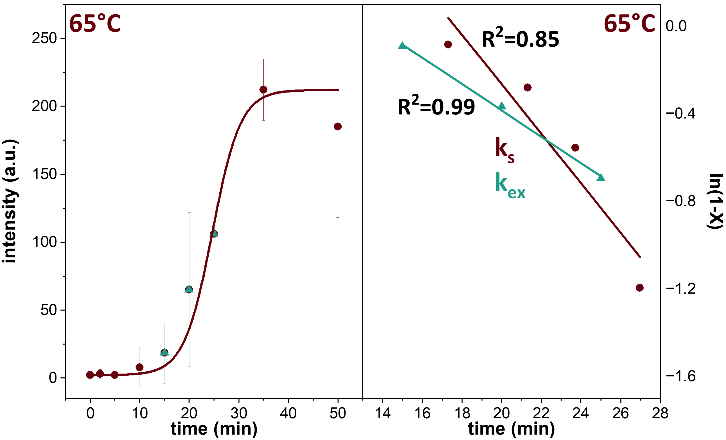
**

**Figure S11.** (left panel) Sigmoidal fit of time-dependent conversion of TCDA to sPDA based on fluorescence emission intensities with λ_em_ = 548 nm, with photopolymerization carried out at 65°C. (right panel) Semilogarithmic plot of conversion versus time. Green markers in each sigmoidal fit graph indicate data points used in semilogarithmic graph to generate fits used to determine k_ex_. The other linear fit determines k_s_.

Experimental data points exhibited some variability, so we compared multiple methods for calculating activation energy. These were both based on the rate of conversion to polymer in the autocatalytic regime, using either experimental data points (Table S2) or equivalent points extracted from the fitted sigmoidal curve (Table S3).

**Table S2**. Display of rate constant k for the respective photopolymerization temperatures, determined from the slope of the semilogarithmic plot of conversion, using experimental data points.

**Table S3**. Rate constant k for the respective photopolymerization temperatures, determined from the slope of the semilogarithmic plot of conversion, using points extracted from the autocatalytic regime of the sigmoidal fits.

To calculate the rate constant k, a semilogarithmic plot of ln k vs. 1/T (K^-1^) is first generated, and the best fit line calculated (Figure S12a-b). The activation energy is then calculated as:

$$lnk=-\frac{Ea}{R}\frac{1}{T}+lnA$$

$$m=slope=-\frac{Ea}{R}$$

where E_a_ = activation energy, R = 8.314 J mol^-1^ K^-1^, and A = frequency factor.


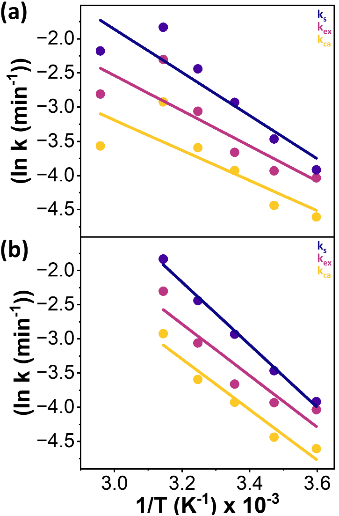


**Figure S12.** Arrhenius plots over temperature ranges (a) 5–65°C and (b) 5–45°C, with linear fits for rate constants k_ca_ (yellow trace), k_ex_ (purple trace), and k_s_ (blue trace).

Arrhenius plots generated using data collected from 5°C–65°C (Figure S12a, Table S4) are similar (within error) across the analysis methods tested; however, R^2^ values are low, primarily due to anomalously fast polymerization at 65 °C in comparison with the other temperatures tested.

Analysis of polymerization restricting values to 5–45°C (Figure S12b, Table S5) produces higher R^2^ values. Calculated values based on t_1/2_ (k_ca_) and experimental slope in autocatalytic region (k_ex_) are similar within error: E_a_(k_ca_) = 7.3 ± 1.0 kcal mol^-1^, E_a_(k_ex_) = 7.5 ± 1.5 kcal mol^-1^, while the value calculated from the sigmoidal curve (k_s_) is slightly higher: E_a_(k_s_) = 9.1 ± 0.5 kcal mol^-1^.

**Table S4**. Slopes from linear fitting of ln k vs. 1/T at 5–65 °C, and calculated activation energies.

**Table S5**. Slopes from linear fitting of ln k vs. 1/T at 5–45 °C, and calculated activation energies.

**Determining the influence of UV irradiation on surface temperature.** Although the Peltier thermoelectric plate was used to establish a setpoint temperature during UV photopolymerization, we also examined the extent to which UV irradiation might further increase the temperature of the HOPG. Adhering a thin piece of HOPG to a thermocouple, we placed the HOPG in contact with a thermally controlled stage set to 35 °C (Figure S13a) to evaluate the temperature sensitivity of the thermocouple. After 50 s of contact, the temperature stabilized at 34.3 °C. In addition, we monitored the temperature of the HOPG briefly after turning off the thermally controlled stage, and we observed a slow decrease in the HOPG temperature matching (±0.3 °C) the temperature reading for the stage. Next, we placed the thin HOPG adhered to the thermocouple onto the Peltier plate inside the environmental chamber, allowing for stable temperature reading. Using the UV lamp, we irradiated the HOPG, measuring the temperature with respect to exposure time (Figure 13b). Over the course of 60 min, the temperature increased by *ca.* 1.5 °C, well under the 10 °C difference between setpoint temperatures utilized in the main manuscript.

**
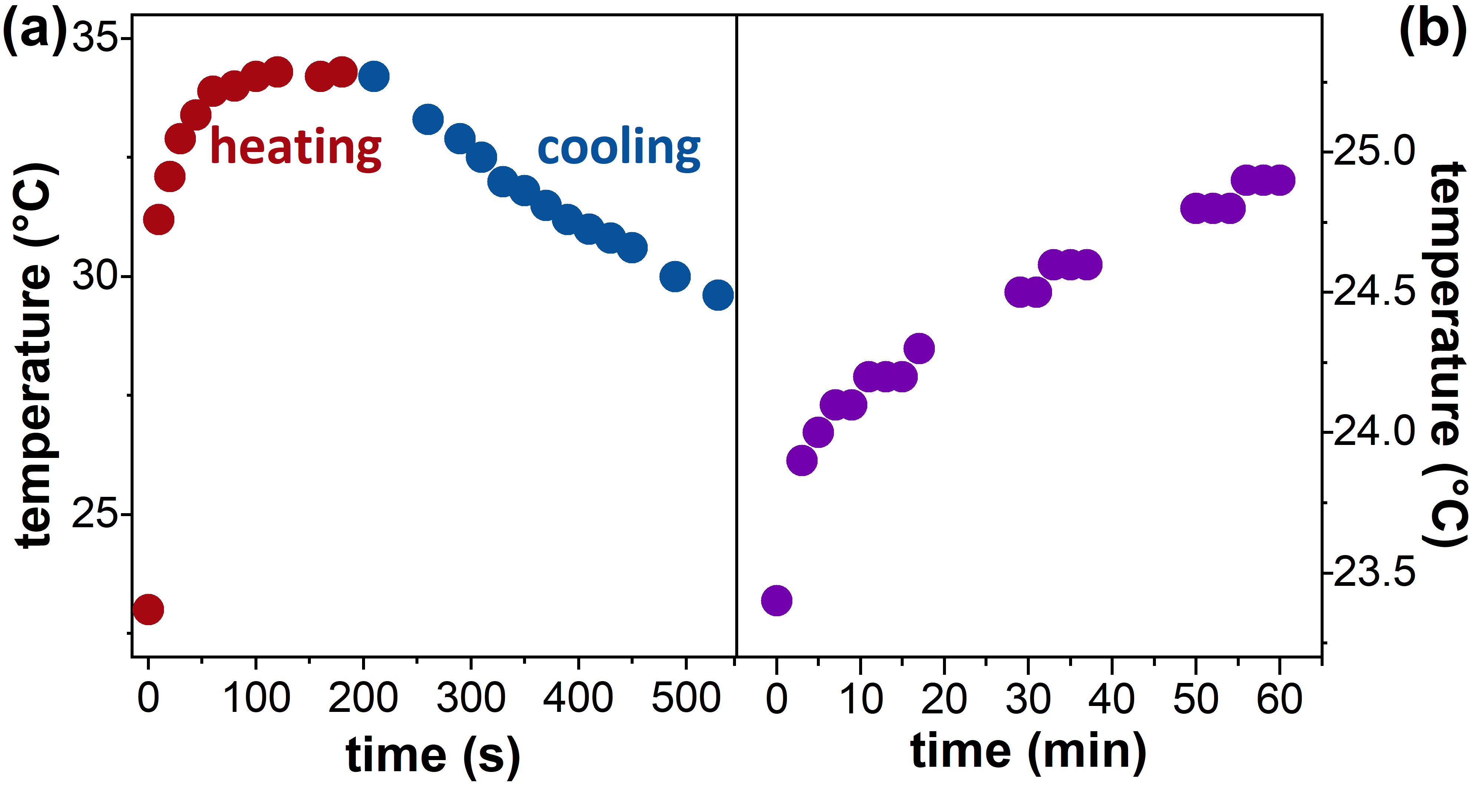
**

**Figure S13.** Time-dependent temperature readings of HOPG adhered to a thermocouple, for (a) HOPG surface in contact with a thermally controlled stage set to 35°C, and (b) HOPG temperature with respect to UV exposure time at laboratory ambient temperature.

**Annealing experiments.** Prior to photopolymerization, samples are thermally equilibrated on the Peltier plate for 30 min. To assess whether annealing of defects during the *equilibration period* might be the primary source of increased polymerization efficiency, we carried out a series of control experiments in which monolayers were placed on the Peltier plate at elevated temperatures (50 °C - 65 °C) for 1 hr followed by steady cooling to 25 °C on the plate, followed by photopolymerization at 25 °C for 50 min (Ann.xx°C-PT25°C, Figure S14a-b). These samples were compared to immediate polymerization at 25 °C without pre-annealing (PT25 °C – 50min), and thermally accelerated photopolymerization (TAP) as described in the manuscript (PT65 °C – 50min, Figure S14 c-d). Subsequently, covalent transfer of sPDA on HOPG to PDMS was performed, and PDA fluorescence emission was analyzed. For these experiments, each condition was calculated based on 4 ROI from a total of 9 samples, with ROIs chosen from in-focus areas. Testing for outliers was achieved using the quartile method previously reported.^17^ Annealing at 50 °C (orange trace) produced essentially the same result as no annealing (blue trace), while annealing at 65 °C produced a moderate increase in polymerization efficiency (red trace, ~70 a.u. vs 40 a.u. for non-annealed). However, a substantially larger increase (~110 a.u. vs 40 a.u. non-annealed) was observed for the samples polymerized at 65 °C, suggesting that carrying out the reaction at elevated temperatures produces impacts distinct from annealing alone.

**
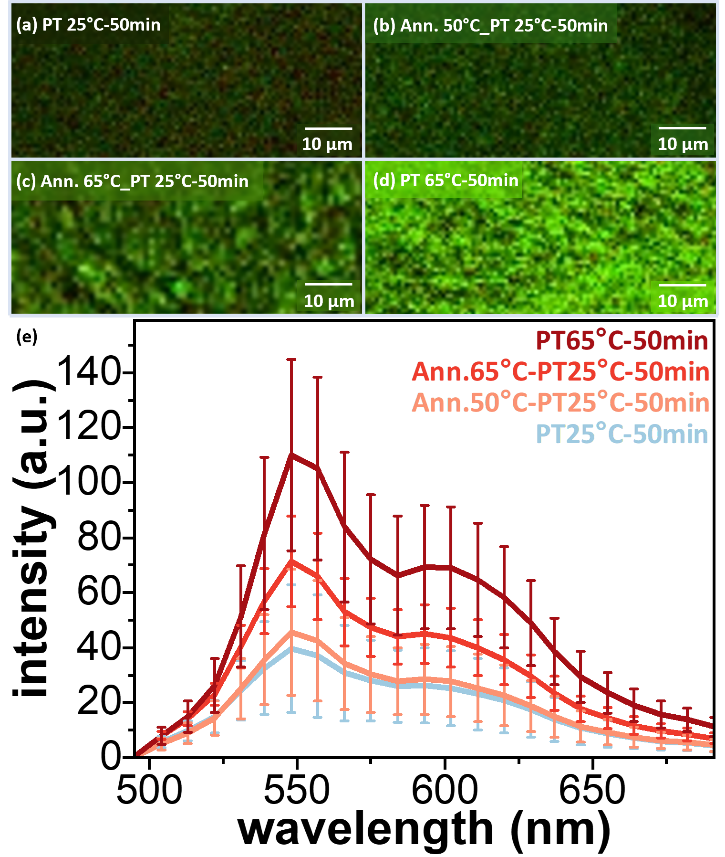
**

**Figure S14.** Confocal images of TCDA/PDMS transferred from TCDA/HOPG after being (a) photopolymerized at set-point temperature of 25 °C, (b) annealed for 1 hr at 50 °C, then polymerized at 25 °C, (c) annealed for 1 hr at 65 °C, then polymerized at 25 °C, and (d) polymerized at 65 °C. (e) Fluorescence spectra for each condition described in (a-d).

**Data analysis for fluorescence timepoints.** The main manuscript presents emission intensities of TCDA monolayers polymerized on HOPG for specified times (0–100 min), then transferred to PDMS. Each data point represents the mean of 9 regions of interest (ROIs) selected from at least 3 samples. In Tables S6 to S10, we tabulate the mean fluorescence intensities (with standard deviations) for each polymerization set-point temperature (5°C‒45°C).

***
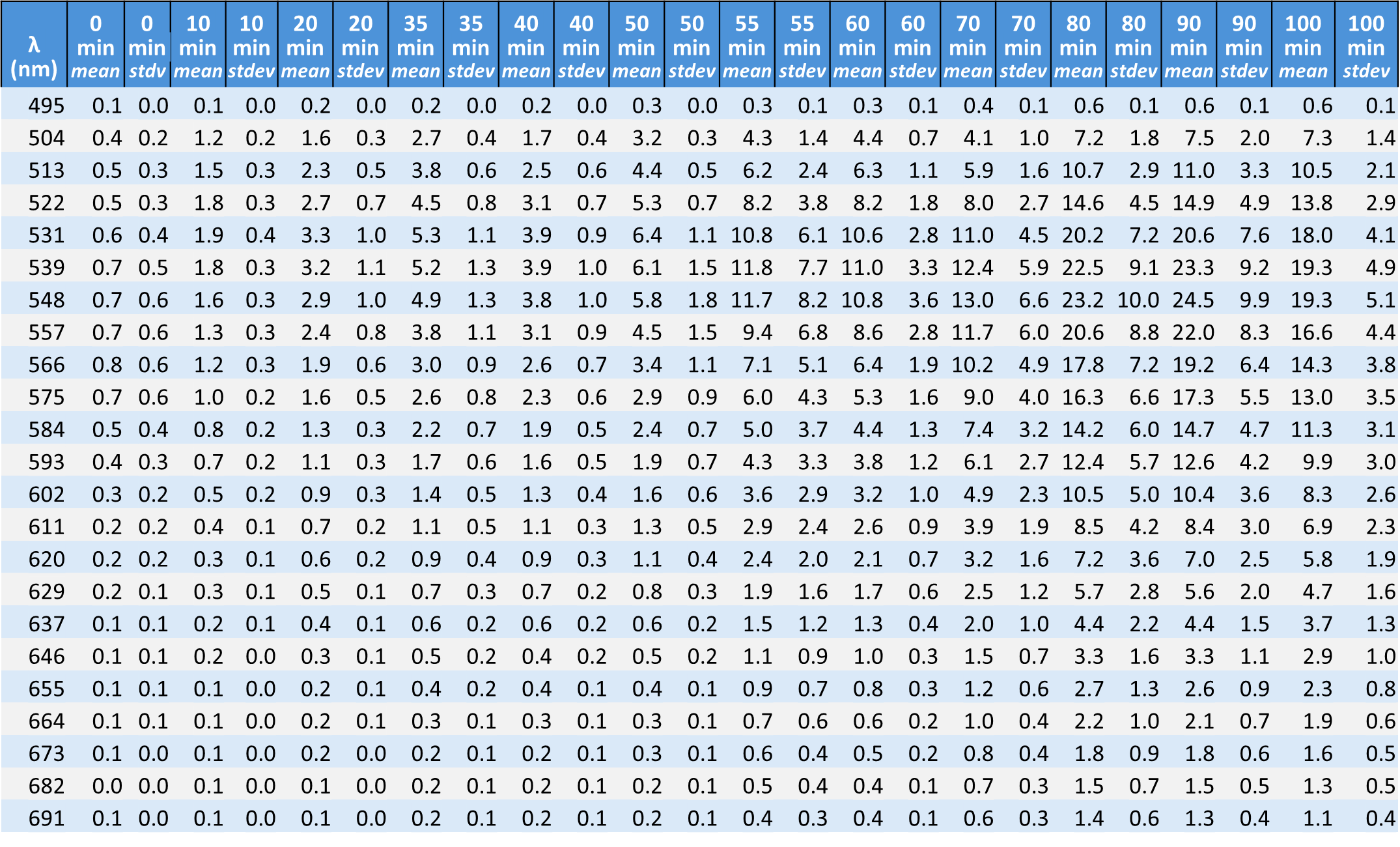
* Table S6**. Mean and standard deviation fluorescence values for regions of interest collected on TCDA/PDMS samples prepared from TCDA/HOPG samples polymerized at a set-point temperature of 5 °C. For each indicated timepoint, 9 ROIs were analyzed from 3 samples.

**
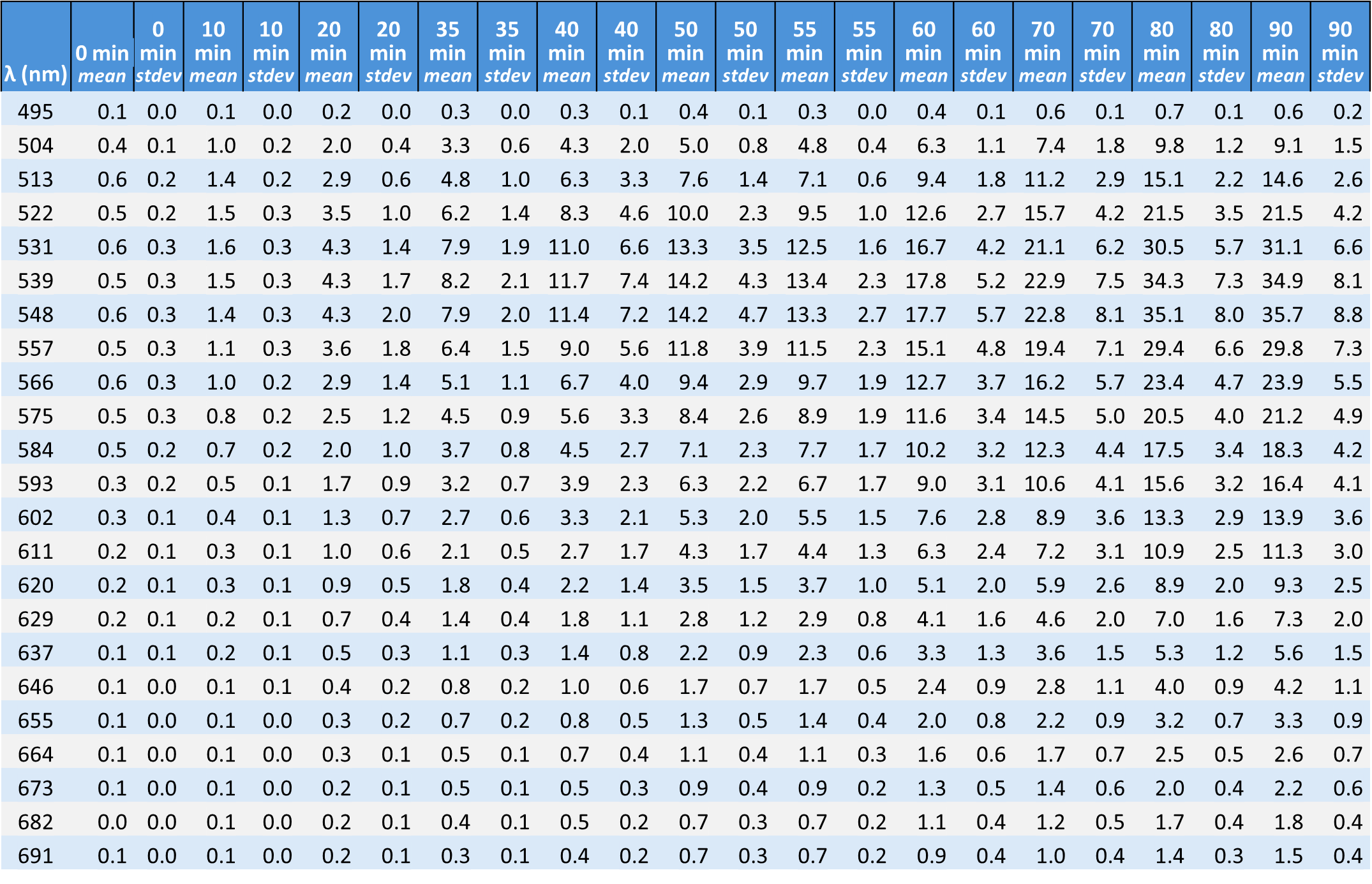
Table S7**. Mean and standard deviation fluorescence values for regions of interest collected on TCDA/PDMS samples prepared from TCDA/HOPG samples polymerized at a set-point temperature of 15 °C. For each indicated timepoint, 9 ROIs were analyzed from 3 samples.

**
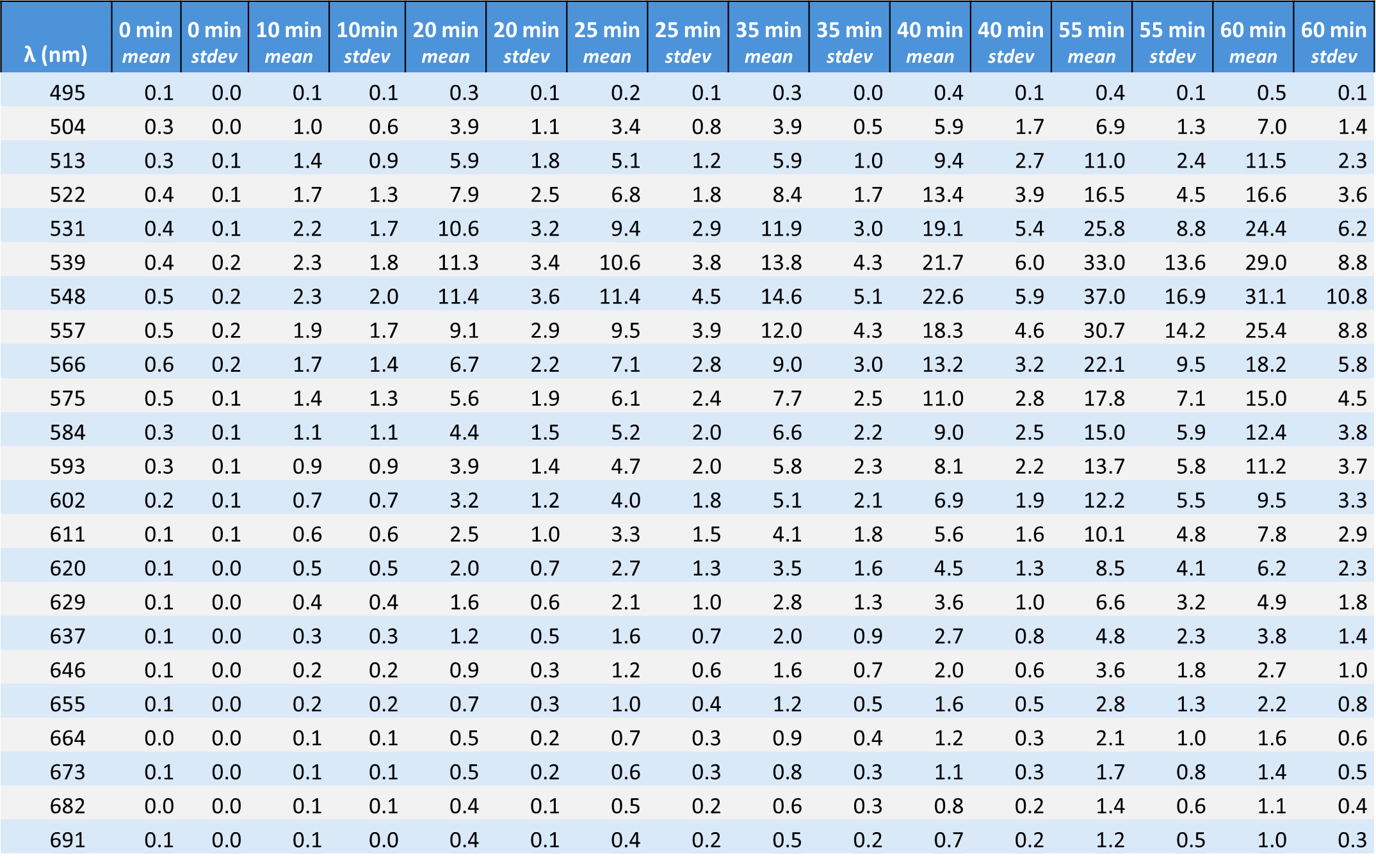
Table S8**. Mean and standard deviation fluorescence values for regions of interest collected on TCDA/PDMS samples prepared from TCDA/HOPG samples polymerized at a set-point temperature of 25 °C. For each indicated timepoint, 9 ROIs were analyzed from 3 samples*.*

**
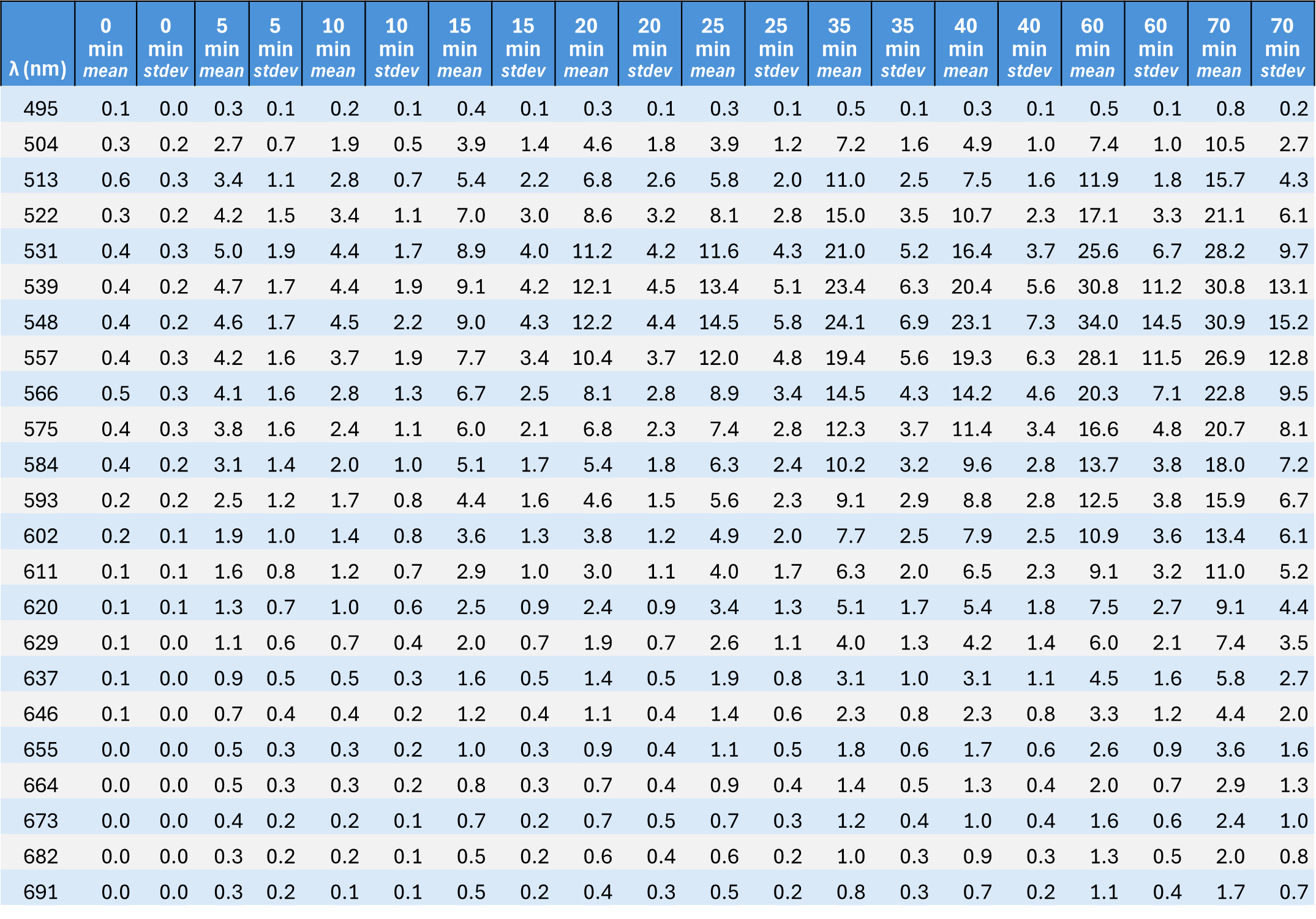
**

**Table S9**. Mean and standard deviation fluorescence values for regions of interest collected on TCDA/PDMS samples prepared from TCDA/HOPG samples polymerized at a set-point temperature of 35 °C. For each indicated timepoint, 9 ROIs were analyzed from 3 samples.

**Table S10**. Mean and standard deviation fluorescence values for regions of interest collected on TCDA/PDMS samples prepared from TCDA/HOPG samples polymerized at a set-point temperature of 45 °C. For each indicated timepoint, 9 ROIs were analyzed from 3 samples.

**Calculation of PDMS transfer probabilities of PDAs.** We used modifications of probabilistic models we developed previously^19^ to evaluate the likelihood of transfer of PDAs based on PDA length. Overall, populations of longer PDAs formed at higher temperatures would be expected to exhibit higher probabilities of transfer individually (in comparison with shorter PDAs) leading to higher fractional transfer as measured by PDA fluorescence emission on PDMS.

The probability of transfer of a polymer with a given degree of polymerization (DP), was calculated using a model that assumes the probability of reaction at each repeat unit of a PDA chain (p_rxn_) is equivalent, and that at least 2 covalent linkages are required in order to transfer a PDA (n≥2), based on our previous experiments. The probability of forming *n* crosslinks to a polymer of length DP, given a specified p_rxn_, is calculated as:

$$P_{n}\left( \mathrm{DP} \right)=C\left( DP, n \right)\times p_{\mathrm{rxn}}^{n}\times\left( 1-p_{\mathrm{rxn}} \right)^{DP-n}$$

In practice, the probability of forming 2 or more crosslinks is calculated as P_n≥2_ = 1 ‒ P_0_ – P_1_. These calculations were used to establish curves of the type shown in Figure 6b of the main manuscript, assuming values of p_rxn_ in the range from 0.005–0.05. The values are then multiplied by the distribution of DP values (such as the model lognormal distribution shown in Figure 6c left panel) to yield the fraction of the monomers in a monolayer that transfers as part of PDAs of length DP (Figure 6c right panel).

To establish the projected fractional transfer for populations of polymers observed and measured experimentally, and the impacts of variations in the distribution of PDA lengths, we then multiplied the length (DP) of each polymer in a population of measured polymer lengths by P_n_(DP). Polymer lengths are included in Tables S11 – S15 below. Example histograms that calculate transfer probabilities are shown in the main manuscript Figure 6c. These values were then used to calculate the fraction of the PDA layer predicted to transfer at each temperature in comparison with predicted transfer for polymerization at 5 °C, as shown in the main manuscript Figure 6d and 6e.

*
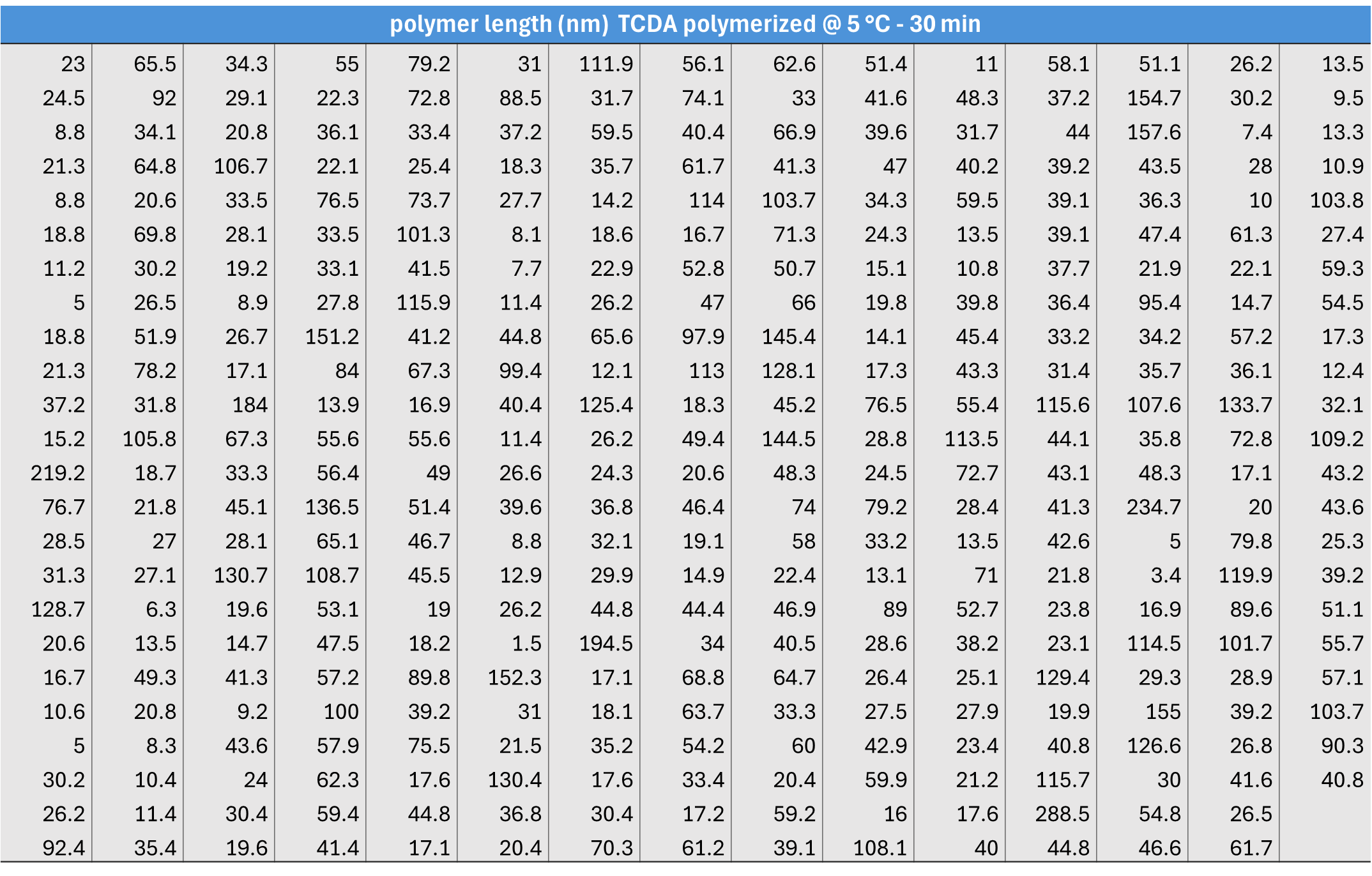
***Table S****11.** Polymer lengths measured in AFM images of TCDA samples polymerized at 5 °C for 30 min.

**

**Table S12.** Polymer lengths measured in AFM images of TCDA samples polymerized at 25 °C for 30 min.

**
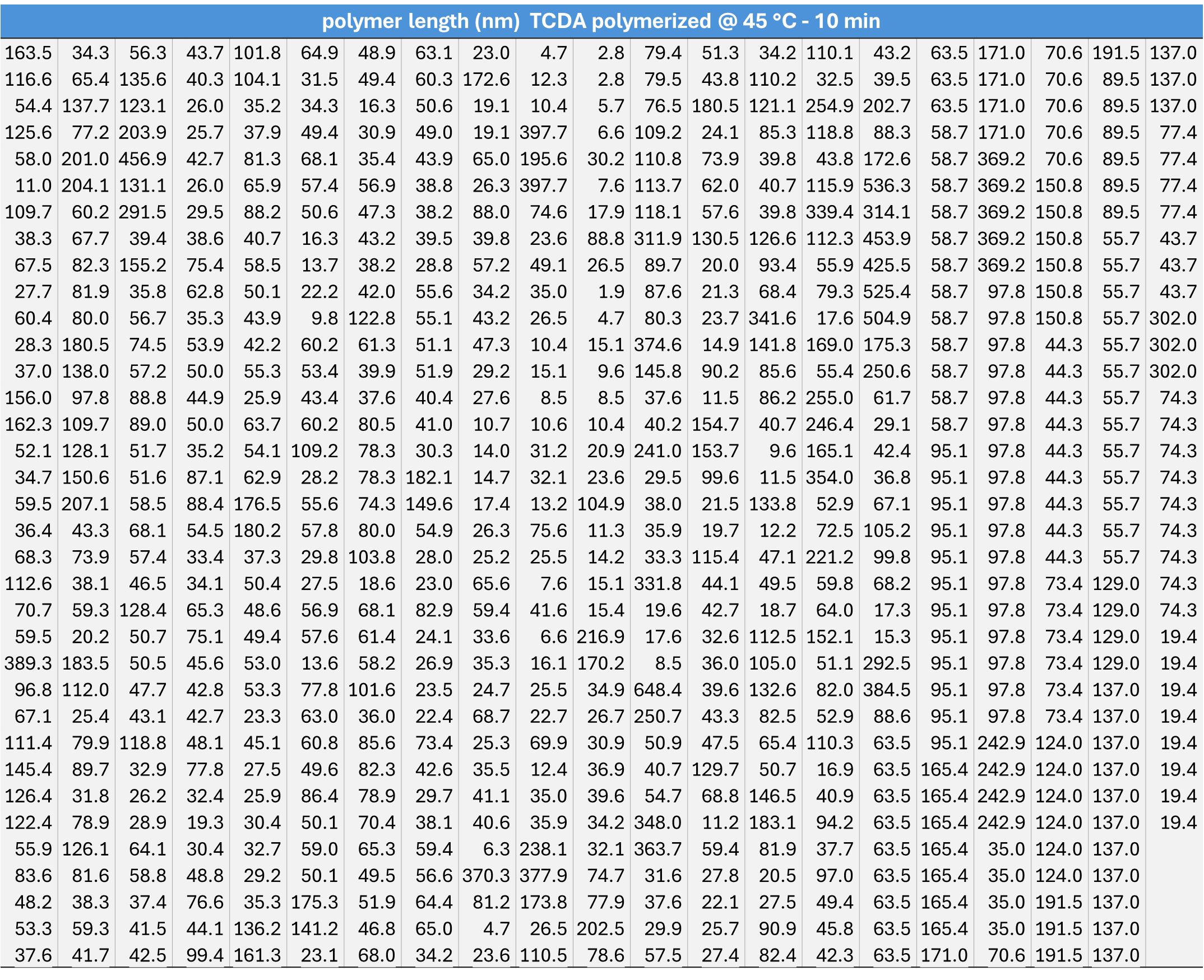
Table S13**. Polymer lengths measured in AFM images of TCDA samples polymerized at 45 °C for 10 min.

**Table S14**. Polymer lengths measured in AFM images of TCDA samples polymerized at 65 °C for 10 min.

**Table S15**. Polymer lengths measured in AFM images of TCDA samples polymerized at 75 °C for 10 min.

**Calculation of average % monomer polymerized.** Calculations based on AFM image area, molecular footprint, DP, and number of polymers visible within the image area were used to estimate the fraction of polymerized monomers in a region. As described in the main manuscript, overall at high polymerization temperatures we observed a larger fraction of polymerized monomers, in agreement with the observed higher PDA fluorescence emission observed in confocal images.

The percentage of monomers polymerized was calculated by first estimating the number of monomers in an AFM image by dividing the image area by the molecular footprint (1.5 nm^2^) of TCDA.

$$total monomers in image region=\frac{\left( image width nm \times image length nm \right)}{\left( 1.5 \frac{\mathrm{nm}^{2}}{\mathrm{monomer}} \right)}$$

Next, the fractional conversion to polymer was calculated as the sum of all polymer lengths (as DP) visible in each AFM image, divided by the number of monomers that would occupy the image area.

$$\% monomers polymerized= \frac{\sum_{i} N_{i}\mathrm{DP}_{i}}{total monomers in image region}\times100\%$$

We additionally calculated the number density of polymers in each image area. This enabled us to compare both the number of initiation events per unit area, as well as the propagation length.

$$number polymers per area= \frac{\sum N_{i}}{\left( image width nm \times image length nm \right)}$$

Tables S16 and S17 below contain the outputs of these calculations for TCDA polymerized at 5 °C for 30 min (Table S11) or 45 °C for 10 min (Table S13).

**Table S16**. Calculated percentage of monomers polymerized with a given region, and number of polymers per unit area, for TCDA polymerized at 5 °C for 30 min.

**Table S17**. Calculated percentage of monomers polymerized with a given region, and number of polymers per unit area, for TCDA polymerized at 45 °C for 10 min.

**Calculation of probability of propagation.** In addition to lognormal fits presented in the main manuscript, we additionally fit histograms of PDA lengths with functions based on the relative probabilities of propagation and termination:

$$f(DP)=\left( DP-1 \right)p^{DP-2}\left( 1-p \right)^{2}$$

in which ‘p’ is probability of a propagation step, and ‘1-p’ is the probability of termination in a given step.^16^ These fits led to calculated propagation probabilities ranging from p(5 °C) = 0.977 to p(65 °C) = 0.991 (see Supporting Information Figure S15). The fits at 25 °C and 65 °C produced low R^2^ values; for the distribution at 25 °C, this was due to larger numbers of short polymers (suggesting the true value of p(25 °C) is somewhat lower than the fitted value), and at 65 °C, this was due to larger numbers of long polymers (suggesting that the true value of p(65 °C) is somewhat higher than the fitted value.


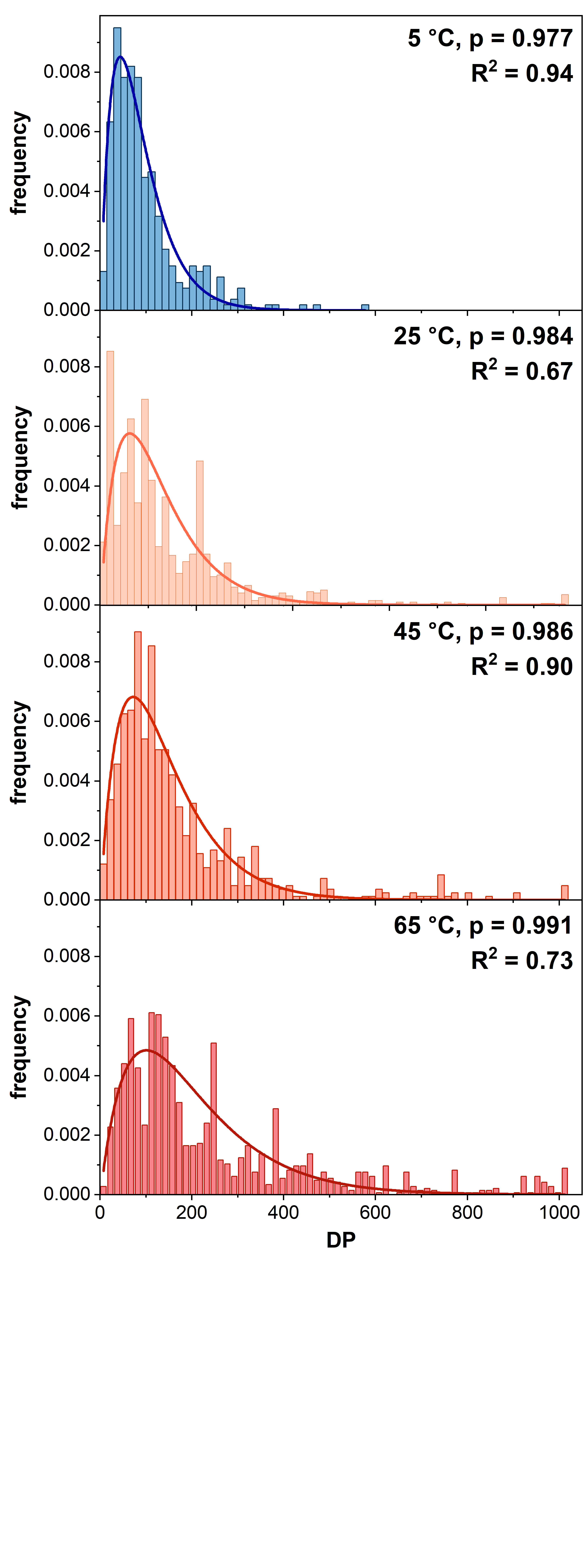


**Figure S15.** Histograms of populations of polymer lengths generated at the stated polymerization temperatures, with curves fitted based on the probability of the propagation step of the reaction.

Next, we revised the calculations presented in Figure 6 to incorporate two factors that would be expected to lead to lower DP values later in the polymerization process: (1) incorporation of point defects in the lattice due to side reactions of photoexcited monomers, which act as termination sites, and (2) the presence of increasing numbers of PDA chains in the lattice, which also act as termination sites.

To model the incorporation of point defects, we treated each calculated probability as a function of time, such that p(t) = p_0_ – at, where p_0_ is the probability of propagation at t = 0 min. In the first set of such models, we assumed that in 100 min (the maximum timepoint used in the manuscript), 1% of monomers were deactivated through side reactions, so a = 0.0001. Since each histogram was acquired at a timepoint after t = 0 min (t_hist_), we calculated p_0_(T) = p_hist_ + a*t_hist_.

Next, a similar set of calculations was performed also taking into account the presence of existing polymers in the lattice as a form of defect that decreases the average DP of polymers formed later in the reaction process. In these calculations, we used the ratios of DP values for polymers formed at each temperature to modify the total probability decrease. For instance, if the maximal probability decrease due to existing PDAs in the monolayer was considered to be 0.015 for polymerization at 5 °C, the probability decrease at 65 °C should be lower at the same percentage conversion, since fewer (but longer) polymers are embedded in the monolayer to act as defects. So the probability of propagation at polymerization temperature T and time t, assuming the probability decrease due to photoexcitation defects as 0.0001/min and the probability decrease due to existing PDAs was 0.015, would be:

$$p\left( T,t \right)=p_{0}\left( T \right)-0.0001t-0.03\left( fractional conversion to polymer at time t \right)(\frac{DP\left( t \right)}{DP\left( 5C \right)})$$

This is shown in the graph in Figure S16 below, with the lowest initial value of p, as well as the largest decreases in p, evident for polymerization at 5 °C (dark blue trace). Traces for polymerization at higher temperatures terminate at the longest timepoint tested experimentally for those temperatures. Within each trace, there is a more rapid decrease in probability evident in the window of time in which the monolayer converts to polymer (e.g. t = 20–30 min, for 65 °C).


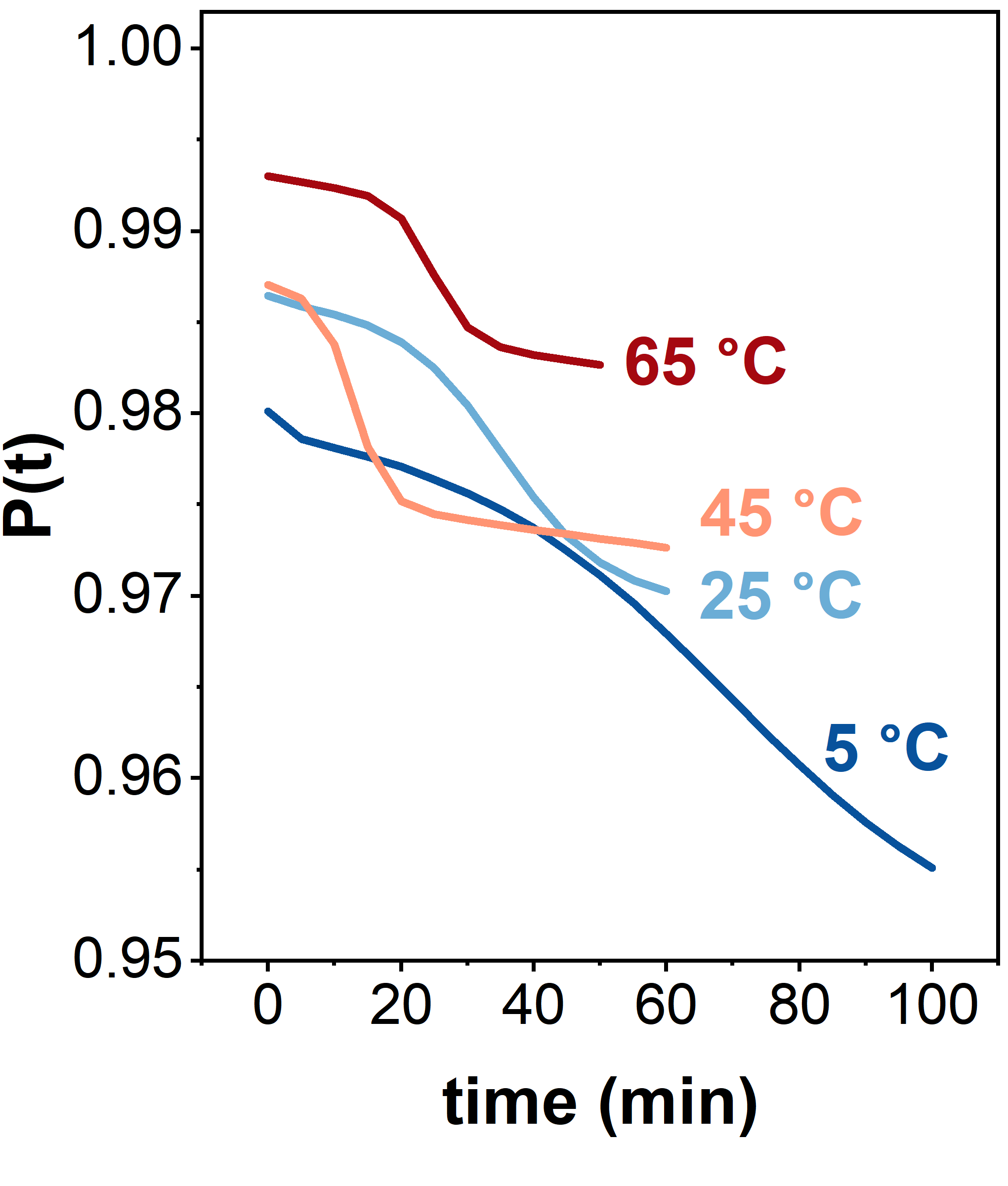


**Figure S16.** Calculated variation in propagation probability with time for each temperature tested in the main manuscript.

Using these p values, we then calculated propagation probabilities every 5 min for each temperature, using these to generate sets of calculated polymer length distributions (such as those shown in Figure 7d of the main manuscript). At each temperature, decreased propagation probability with time decreases DP. These distributions were then multiplied by the fraction of total polymerization that occurred during that 5-min interval, based on kinetics data in main manuscript Figure 3g, and a curve predicting transfer efficiency for polymers at each DP value similar to those in main manuscript Figure 6b, to calculate the amount of polymer transfer occurring within each 5-min period at each temperature. These curves were summed through the range of timepoints measured at each temperature (Figure 7f) then multiplied by fractional transfer curves to estimate total polymer transfer at each temperature, as shown in main manuscript Figure 7g.

**Molecular models of TCDA dynamics at temperatures from 5 °C to 165 °C.**

We carried out molecular dynamics simulations to further examine factors contributing to the observed temperature dependence of the reaction. First, to examine overall monolayer stability prior to polymerization, we calculated 5 ns dynamics at from 5 °C to 165 °C in 20 °C intervales, using the same built systems as a starting point. Model systems were generated using Schrodinger Maestro, and dynamics were carried out using the Desmond software package within the Schrodinger interface. Desmond generates a user-specified parallelepiped box around the model system; this global cell is used to establish periodic boundary conditions for dynamics.

An initial model TCDA monolayer was created by generating 4 rows of 35, 34, or 33 molecules each on a layer of graphene, as shown in Figure S17 (which illustrates the 35-molecule-per-row model). In the initial model, prior to relaxation, molecules are slightly overpacked as shown in Figure S16a, to ensure they do not overhang the edge of the graphite sheet, the dimensions of which are used to ensure consistency of the global cell parameters. The Desmond System Builder utility was used to create a molecular dynamics input model using no solvent, box size buffer parameters of 0 Å at left and right edges of the model (in the orientation shown in the figure), 0.2 Å buffer at the top and bottom edges, and 10 Å buffer perpendicular to the image plane, for global cell dimensions of 124.6 Å × 161.3 Å × 28.8 Å. These parameters were chosen to create a unit cell that nearly approximates the behavior of a monolayer on a large sheet of graphite. The 0.2 Å buffer at the top and bottom edges was required to accommodate the aligned placements of terminating hydrogens at the top and bottom edges of the model; hydrogens on the left and right edges are staggered, permitting use of the 0 Å buffer. These models were used as inputs to Desmond Molecular Dynamics runs, with a typical simulation time of 5.0 ns, energy recording intervals of 5 ps, trajectory recording intervals of 20 ps, the NVT ensemble class, and a specified temperature from 278.15 to 438.15 K. Simulations were carried out with the graphene sheet constrained with a force constant of 100 kJ/mol Å. As the system is relaxed prior to dynamics, the molecules expand through the connected left and right edges of the box, as shown in Figure S17b; alkyl chain segments that appear to be freestanding are in contact with the opposing edge of the graphene sheet, through the box edge. The use of 35 molecules per row create an alkyl chain spacing of 4.61 Å after relaxation; 34 and 33 molecules per row create alkyl chain spacings of 4.74 Å and 4.89 Å, respectively. The spacing generated by the 34-molecule model most closely resembles spacings reported previously based on STM experiments by others,^20^ and the spacing if chains are allowed to relax without constraints on a larger modeled graphene sheet.


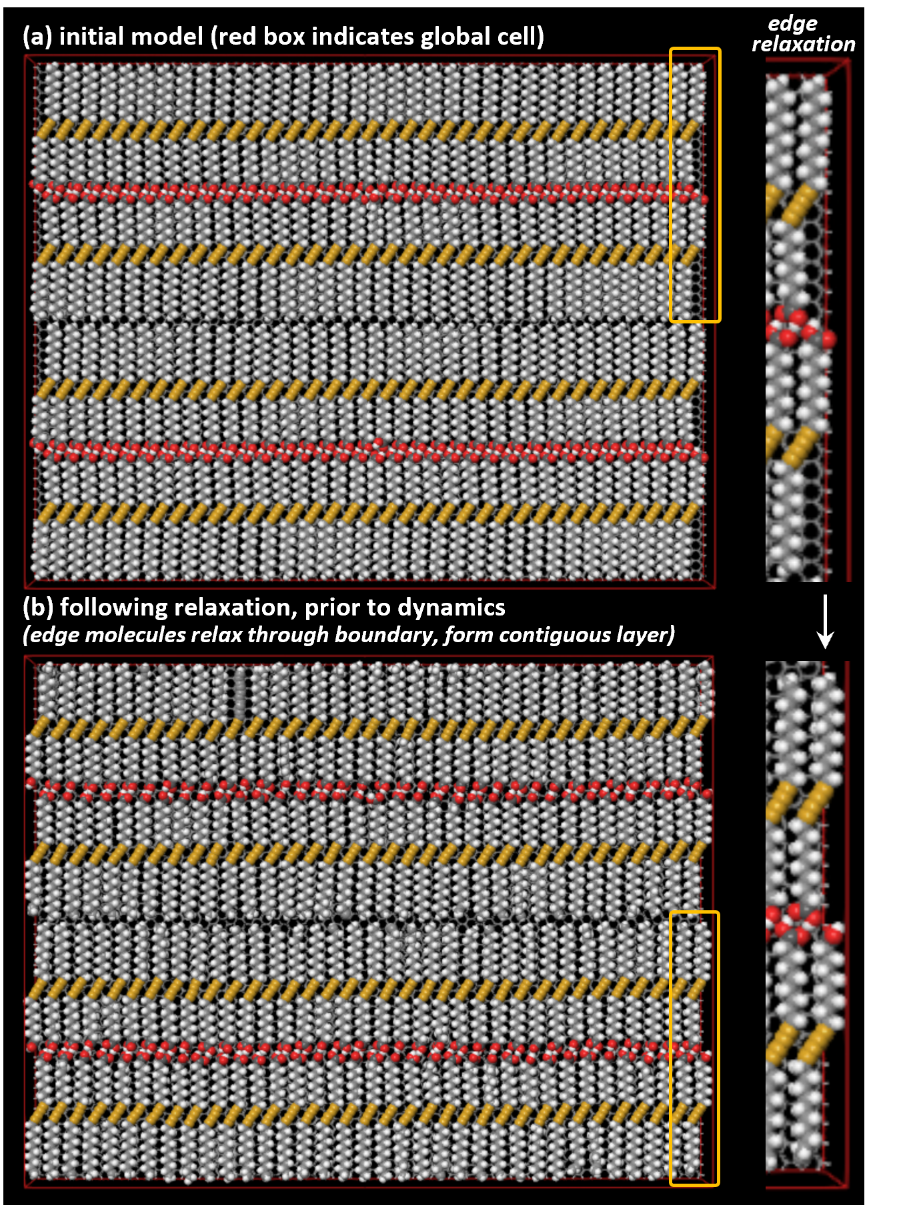


**Figure S17.** (a) Molecular model of TCDA monolayer on graphene sheet, prior to relaxation. Red lines indicate global cell boundaries. Inset to right illustrates molecules slightly overpacked in order for both segments of alkyl chain to rest completely within edge of graphene sheet. (b) Same model following relaxation but prior to dynamics. Inset illustrates apparent molecular overhang at edge of graphene sheet. These segments occupy the apparently vacant surface of graphene on left edge of sheet, forming a contiguous monolayer that undergoes coupled dynamics.

To examine monolayer stability in comparison with prior DSC studies of straight-chain alkanes on expanded HOPG, we carried out simulations across a range of temperatures up to 155 °C, using the 4.7 Å spacing model (Figure S18). Initially, we performed 5 ns simulations with 1.2 ps energy calculation steps and 5 ps trajectory recording steps, at intervals of 20 °C starting at 5 °C. Based on the lowest temperatures at which small defects appeared in the monolayer, we carried out additional simulations at 10 °C intervals, for 5 ns time periods. In these calculations, we observed very limited additional disordering of individual alkyl chains up to temperatures of 115 °C (Figure S18h); at lower temperatures, parts of the terminal alkyl chain segment temporarily break contact with the graphene sheet, while at 115 °C, occasionally a terminal chain segment will completely lose contact with the graphene (e.g. to form contact with the upper face of the monolayer). Persistent defects appeared for simulations at 125 °C (Figure S18i); typically these begin with translation of a COOH-bonded dimer perpendicular to the lamellar axis, creating a defect that extends across both molecular rows. Interestingly, although DSC studies of multilayers of simple alkanes in expanded HOPG observe thermal transitions consistent with formation of a rotator phase near the bulk melting point of the monomer^21-22^ (which here would be ~52 °C), our models do not suggest such a transition occurs for TCDA. Close to the melting transition, individual alkyl chain segments do occasionally rotate (visible in Figure S18f,g), but in aggregate, the chains retain their orientation with the carbon backbone zig-zag parallel to the graphene surface.


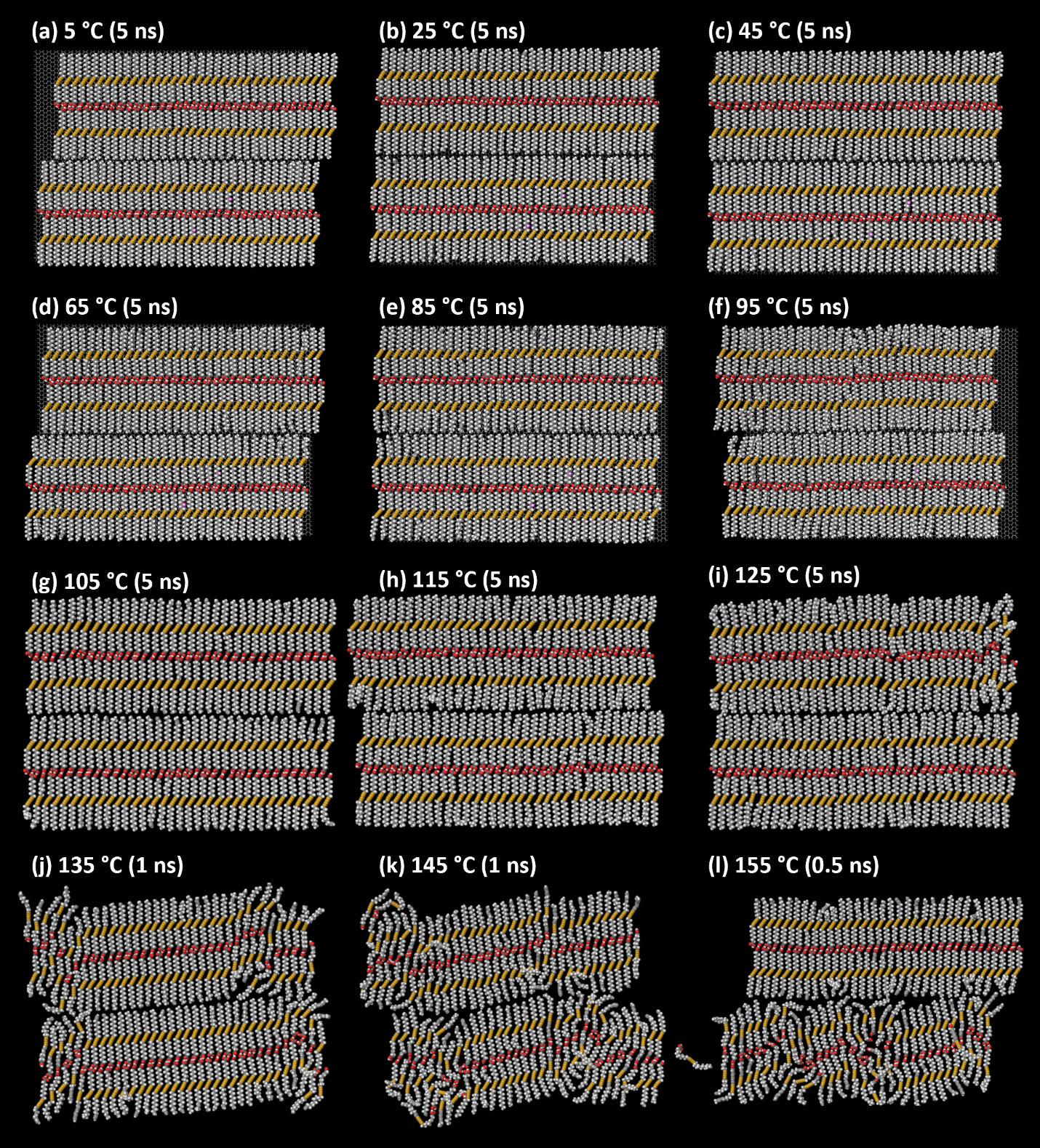


**Figure S18.** Molecular model of TCDA monolayer on graphene sheet with 34 molecules per row (4.7 Å alkyl chain repeat distance) after dynamics carried out for the stated time interval at (a) 5 °C, (b) 25 °C, (c) 45 °C, (d) 65 °C, (e) 85 °C, (f) 95 °C, (g) 105 °C, (h) 115 °C, (i) 125 °C, (j) 135 °C, (k) 145 °C, and (l) 155 °C. Note that molecules that appear to lie off the edge of the graphene sheet have extended into the next instance of the global cell and occupy the apparently vacant region of the graphene. Models were visualized in this way in order to illustrate the nanoscale collective motion of rows of molecules that occurs at the nanosecond timescale.

For comparison, we also carried out calculations on the 33- and 35-chain-per-row models. For the 33-chain-per-row model (Figure S19), the initial 1.2 ns simulations illustrated that, near room temperature, the molecules did not expand to fill the full surface area of the global cell, leaving a few-Ångstrom gap, typically bridged by a pair of H-bonded COOH headgroups, which appear uncoupled at the left and right edges of models since their bonded partner is on the opposite edge of the global cell (see for example Figure S19a,b). Because of this small gap at the left/right edges, the model is likely to exhibit dynamics similar to the edges of molecular domains, and/or small molecular domains (similar to the graphene sheet size of 12 nm × 16 nm). At 45 °C, individual molecules at the edges of the model become more mobile (Figure S19c), while at 55 °C and 65 °C (Figure S19d,e), defects emerged within the domain structure. Interestingly, at 75 °C and 85 °C (Figure S19f), the alkyl chains do begin to expand laterally, associated with more frequent alkyl chain rotations. At 105 °C (Figure S19g), defects rapidly emerge and persist through the end of the simulation. In the context of experiments, we hypothesized that this would mean that the molecules around defects and domain edges are expected to become more mobile at slightly elevated temperatures (*ca.* 45 °C) while small molecular domains may become unstable at further elevated temperatures (65‒85 °C). Conversely, in the 35-chain-per row model, greater thermal stability was observed, with limited defect formation up to 125 °C (Figure S20).


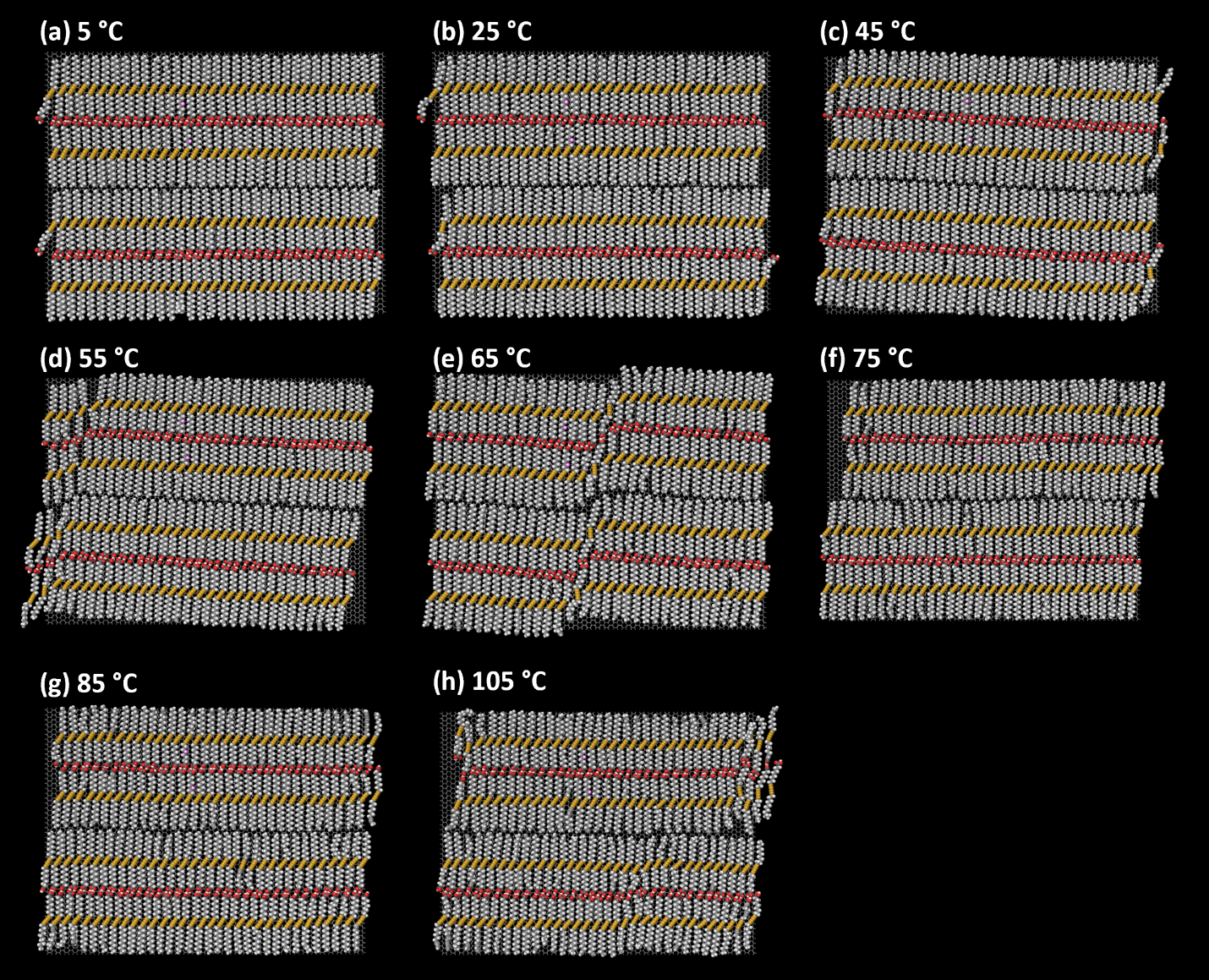


**Figure S19.** Molecular model of TCDA monolayer on graphene sheet with 33 molecules per row (4.9 Å alkyl chain repeat distance) after either 1 ns dynamics carried out at (a) 5 °C, (b) 25 °C, (c) 45 °C, (d) 55 °C, (e) 65 °C, (f) 75 °C, (g) 85 °C, (h) 105 °C. Note that molecules that appear to lie off the edge of the graphene sheet have extended into the next instance of the global cell and occupy the apparently vacant region of the graphene. Models were visualized in this way in order to illustrate the nanoscale collective motion of rows of molecules that occurs at the nanosecond timescale.


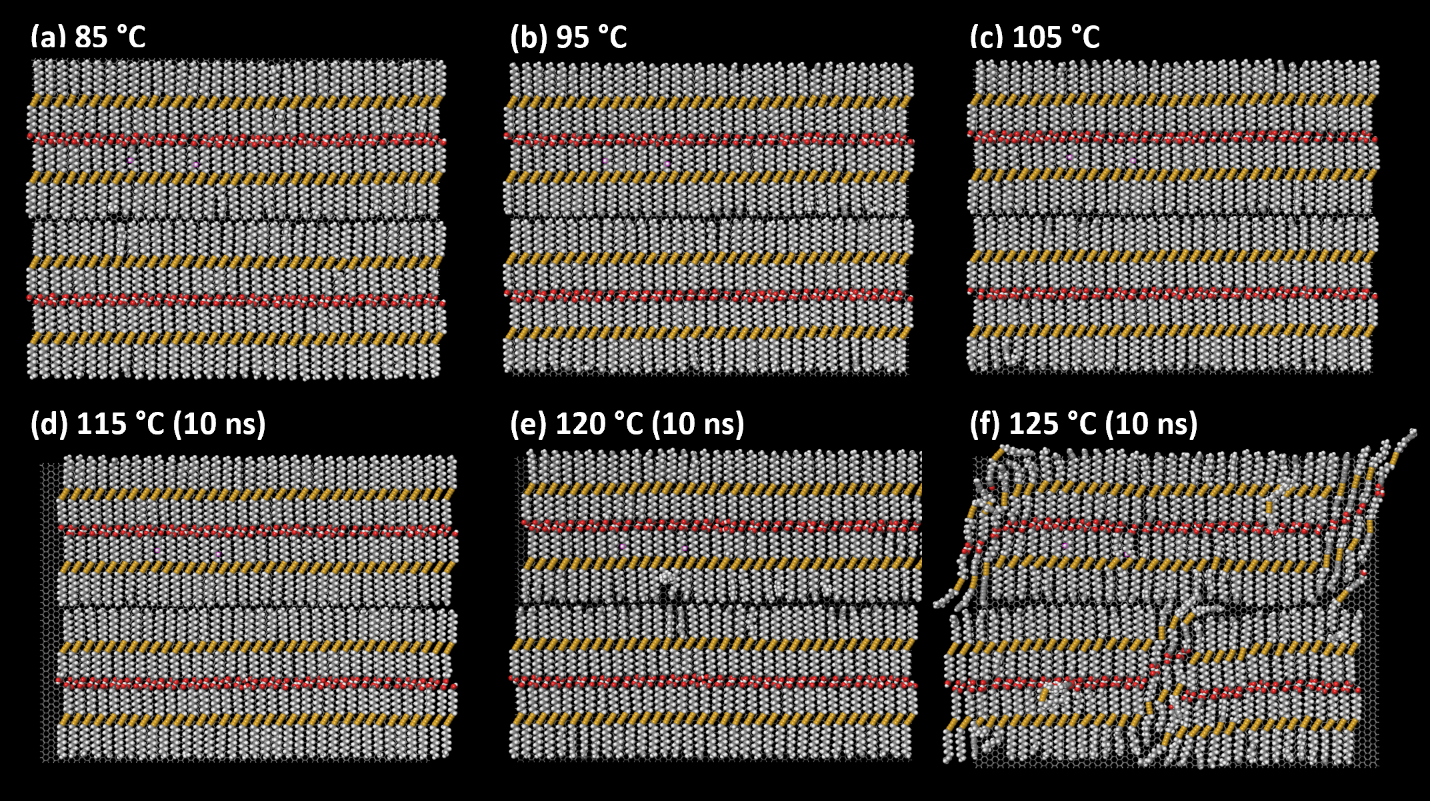


**Figure S20.** Molecular model of TCDA monolayer on graphene sheet with 35 molecules per row (4.6 Å alkyl chain repeat distance) after either 5 or 10 ns dynamics carried out at (a) 85 °C, (b) 95 °C, (c) 105 °C, (d) 115 °C, (e) 120 °C, or (f) 125 °C. Note that molecules that appear to lie off the edge of the graphene sheet have extended into the next instance of the global cell and occupy the apparently vacant region of the graphene. Models were visualized in this way in order to illustrate the nanoscale collective motion of rows of molecules that occurs at the nanosecond timescale.

Next, we examined the temperature dependence of dynamics of polymers embedded in TCDA monolayers, as well as impacts of the polymer on surrounding monomers. Models were constructed beginning from the 34-molecule-per-row system, by replacing 12 monomers in one of the central rows with a 12-repeat-unit polymer, then iteratively minimizing sections of the surrounding structure to re-establish hydrogen bonded dimers and ensure there were no directly overlapping sections of molecules. Models were also subjected to a relaxation phase after the initial Desmond system was built and prior to initiation of dynamics. Figure S21 shows the same model after 5 ns or 1 ns dynamics at the stated temperature; the 1 ns time period is shown if the monolayer is already clearly disordered after that interval, emphasizing the more rapid disordering process.

Narrowing of polymerized lamellae due to rehybridization of the diacetylenes to form the polydiacetylene is visible as a vacancy in the center of some models (*e.g.* in Figure S21a,c); in the other models, the hydrogen bonded opposing monomers shift downward, so the vacancy occupies the top edge of the model unit cell (*e.g.* in Figure S21b,d). As described in the main manuscript, defects in molecular shape complementarity between ends of the PDA and the adjacent monomers result in defect formation at somewhat lower temperatures (105 °C, Figure S21g) than for monomer-only models, and the initial defects in the model typically occur due to slippage of the pair of COOH-dimerized monomers at one end of the PDA (most often adjacent to the C14 end of the PDA, on the left in models in Figure S21). At higher temperatures, interestingly, the PDA and opposing COOH-bonded monomers are often the last remaining ordered section of the model (*e.g.* in Figure S21i), since the PDA is somewhat rigid (persistence length ~15 nm in solution), and acts to retain some degree of local ordering.

*
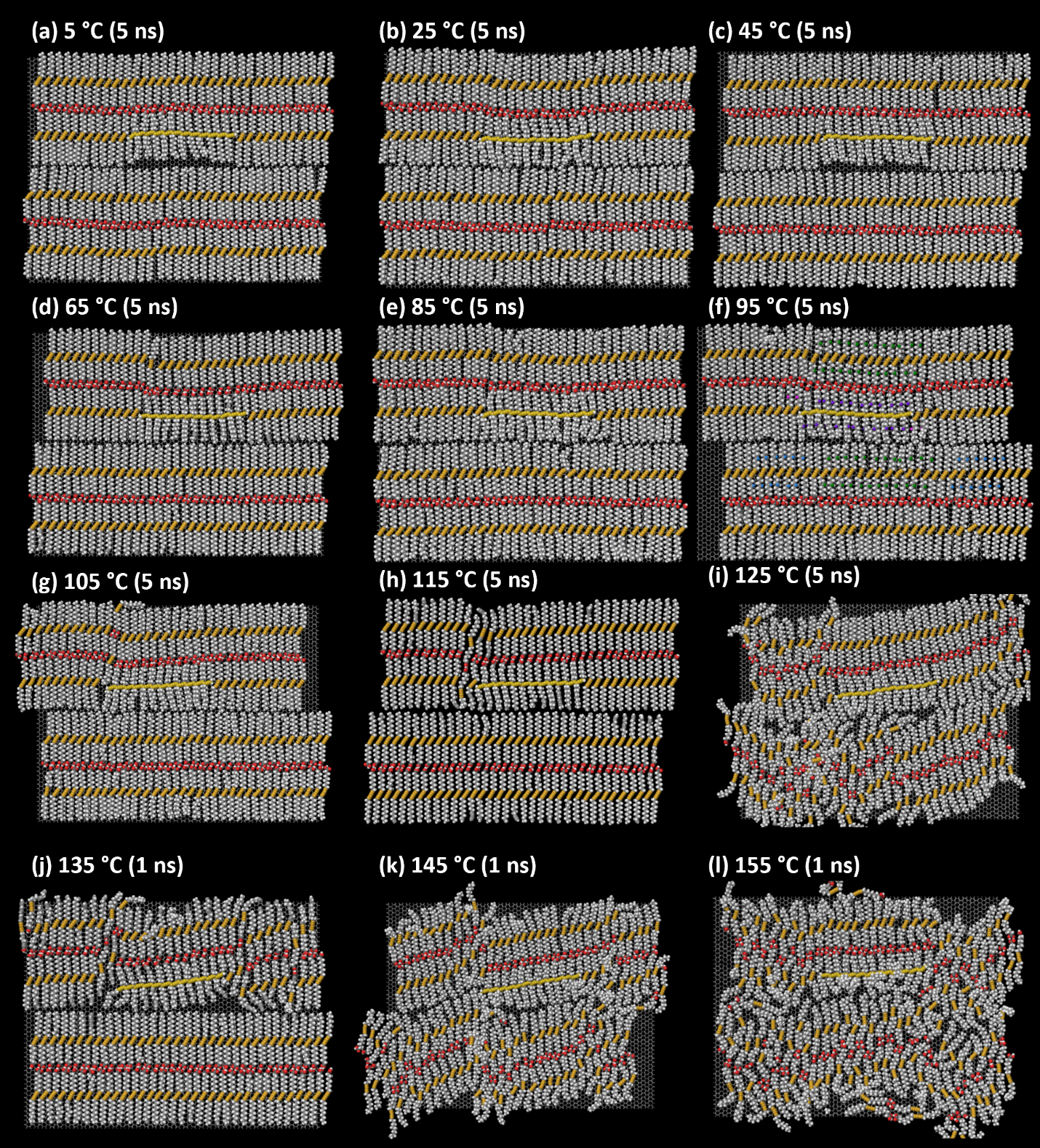
*

**Figure S21.** Molecular model of TCDA monolayer on graphene sheet with 34 molecules per row (4.7 Å alkyl chain repeat distance), in which 12 monomers have been removed and replaced with a 12-unit PDA oligomer. Panels show the model after dynamics carried out for the stated time interval at (a) 5 °C, (b) 25 °C, (c) 45 °C, (d) 65 °C, (e) 85 °C, (f) 95 °C, (g) 105 °C, (h) 115 °C, (i) 125 °C, (j) 135 °C, (k) 145 °C, and (l) 155 °C. Note that molecules that appear to lie off the edge of the graphene sheet have extended into the next instance of the global cell and occupy the apparently vacant region of the graphene. Models were visualized in this framework in order to illustrate the nanoscale collective motion of rows of molecules that occur at the nanosecond timescale.

As discussed in the main manuscript, for the PDA backbone structure, the larger lattice constant along the molecular row direction decreases interactions between adjacent alkyl chain segments, leading to pronounced chain rotation at all temperatures simulated in the course of this work. In the model shown in Figure S22a, several alkyl chain segments of the PDA adopt a conformation with the carbon chain zig-zag perpendicular to the plane of the graphene. In contrast, the modestly tighter packing of the alkyl chains in rows of monomers tends to limit alkyl chain rotation. To quantify the extent to which chain rotation varied both based on the type of chain segment and the temperature at which dynamics calculations were carried out, we selected sets of equivalent hydrogen atoms on adjacent chain segments (color-coded and labeled in Figure S22a), and extracted radial distribution functions (RDFs) for those groups of atoms from Desmond dynamics trajectories, using built-in function calls in the Desmond software. RDFs are shown for PDA and adjacent monomer proximal and terminal segments in the main manuscript. Here, we also show the opposing monomers and other monomers (not directly in contact with the polymer). Overall, as described in the main manuscript, frequent rotation is only observed for the PDA alkyl chains (left panels), as indicated by the presence of a large peak between 2 and 3 Å. Terminal segments of adjacent monomers also exhibit modest amounts of rotation at the highest temperatures graphed (85‒105 °C), but rotation is very limited for both opposing monomers and monomers not in contact with the PDA.


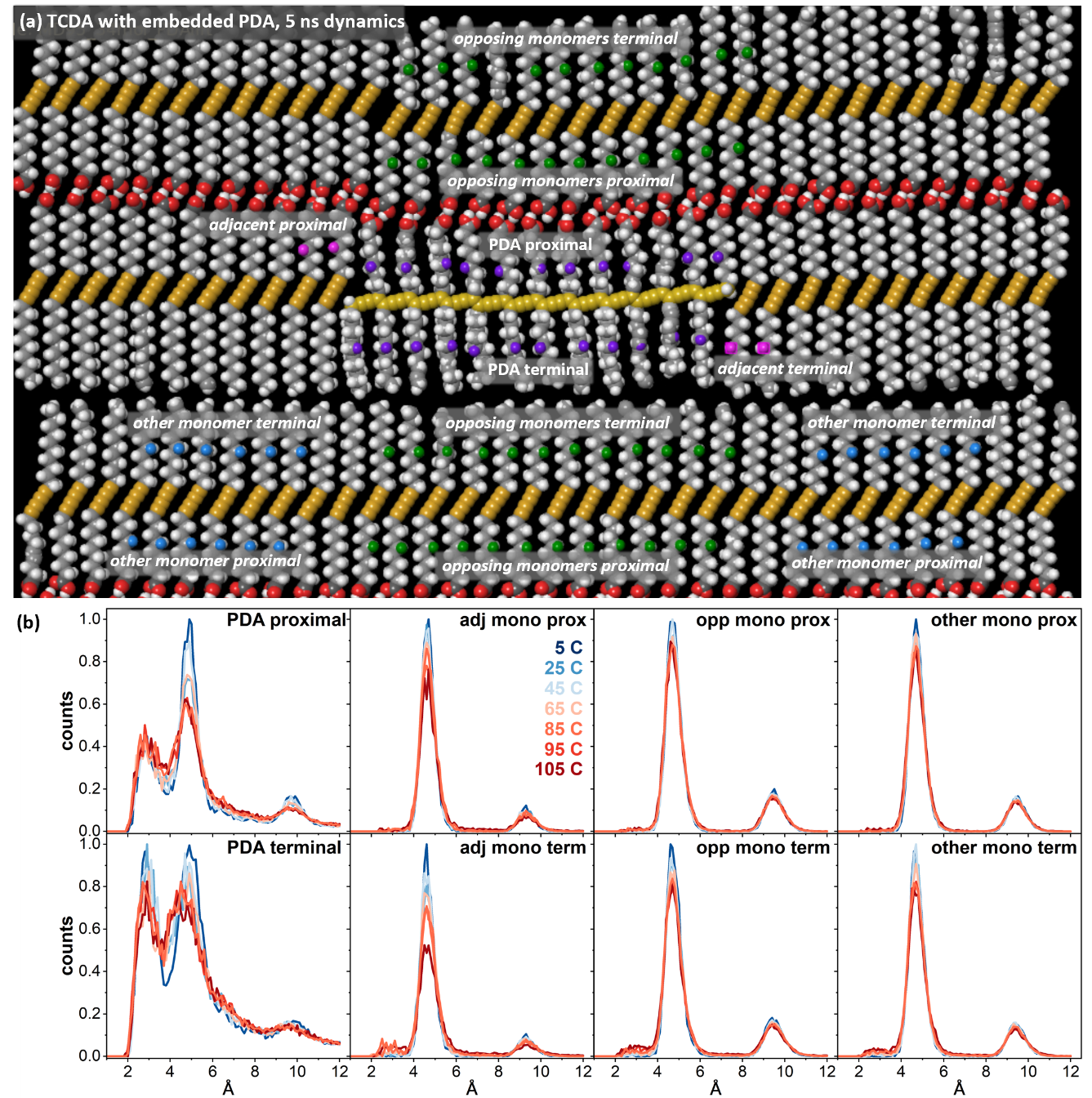


**Figure S22.** (a) Enlarged model from Figure S20f, representing a partially polymerized TCDA monolayer after 5 ns dynamics at 95 °C. Sets of atoms colored purple, pink, green, and blue represent sets of hydrogen atoms used to construct RDFs in panel (b). (b) RDFs for the sets of atoms color-coded and labeled in panel (a), for trajectories from 5 to 105 °C.

REFERENCES CITED

1. Bang, J. J.; Rupp, K. K.; Russell, S. R.; Choong, S. W.; Claridge, S. A. Sitting Phases of Polymerizable Amphiphiles for Controlled Functionalization of Layered Materials. *J. Am. Chem. Soc* **2016,** *138*, 4448-4457.

2. Davis, T. C.; Bang, J. J.; Brooks, J. T.; McMillan, D. G.; Claridge, S. A. Hierarchical Noncovalent Functionalization of 2D Materials by Controlled Langmuir-Schaefer Conversion. *Langmuir* **2018,** *34*, 1353-1362.

3. Bang, J. J.; Porter, A. G.; Davis, T. C.; Hayes, T. R.; Claridge, S. A. Spatially Controlled Noncovalent Functionalization of 2D Materials Based on Molecular Architecture *Langmuir* **2018,** *34*, 5454-5463.

4. Davis, T. C.; Bechtold, J. O.; Shi, A.; Lang, E. N.; Singh, A.; Claridge, S. A. One Nanometer Wide Functional Patterns with a Sub-10 Nanometer Pitch Transferred to an Amorphous Elastomeric Material. *ACS Nano* **2021,** *15*, 1426-1435.

5. Hayes, T. R.; Bang, J. J.; Davis, T. C.; Peterson, C. F.; McMillan, D. G.; Claridge, S. A. Multimicrometer Noncovalent Monolayer Domains on Layered Materials through Thermally Controlled Langmuir-Schaefer Conversion for Noncovalent 2D Functionalization. *ACS Appl. Mater. Interf.* **2017,** *9*, 36409-36416.

6. Hayes, T. R.; Lang, E. N.; Shi, A.; Claridge, S. A. Large-Scale Noncovalent Functionalization of 2D Materials through Thermally Controlled Rotary Langmuir-Schaefer Conversion. *Langmuir* **2020,** *36*, 10577-10586.

7. Chance, R. R.; Patel, G. N. Solid-State Polymerization of a Diacetylene Crystal - Thermal, Ultraviolet, and Gamma-Ray Polymerization of 2,4-Hexadiyne-1,6-Diol Bis-(Para-Toluene Sulfonate). *J. Polym. Sci., Part B: Polym. Phys.* **1978,** *16*, 859-881.

8. Baughman, R. H.; Chance, R. R. Theory of Single-Phase Solid-State Polymerization Reactions. *J. Chem. Phys.* **1980,** *73*, 4113-4125.

9. Patel, G. N.; Chance, R. R.; Turi, E. A.; Khanna, Y. P. Energetics and Mechanism of Solid-State Polymerization of Diacetylenes. *J. Am. Chem. Soc.* **1978,** *100*, 6644-6649.

10. Bloor, D.; Ando, D. J.; Hubble, C. L.; illiams, R. L. Raman Spectroscopic Studies of the Solid-State Polymerization of Diacetylenes - 2. Thermal Polymerization of 1,6-Di-P-Methoxybenzene-Sulfonyloxy-2,4-Hexadiyne. *Journal of polymer science. Part A-2, Polymer physics* **1980,** *18*, 779-791.

11. Enkelmann, V. The Solid-State Polymerization, Physical Properties, and Crystal Structures of Diacetylene Mixed Crystals. *Makromol. Chem.* **1983,** *184*, 1945-1955.

12. Orczyk, M.; Pater, E.; Sworakowski, J. Kineics of Polymerization of Mixed Diacetylene Single Crystals Determined from Dielectric Measurements. *Makromol. Chem.* **1992,** *193*, 1135-1146.

13. Kollmar, C.; Sixl, H. Reaction Kinetics of the Solid State Polymerization in Diacetylene Crystals. *J. Chem. Phys.* **1987,** *87*, 5541-5543.

14. Sixl, H., Spectroscopy of the Intermediate States of the Solid State Polymerization Reaction in Diacetylene Crystals. In *Polydiacetylenes*, Springer Berlin: Heidelberg, 2005; pp 49-90.

15. Alekseev, A. S.; Viitala, T.; Domnin, I. N.; Koshkina, I. M.; Nikitenko, A. A.; Peltonen, J. Polymerization of Modified Diacetylenes in Langmuir Films. *Langmuir* **2000,** *16*, 3337-3344.

16. Takajo, D.; Sudoh, K. Mechanism of Chain Polymerization in Self-Assembled Monolayers of Diacetylene on Graphite. *Langmuir* **2019,** *35*, 2123-2128.

17. Shi, A.; Villarreal, T. A.; Singh, A.; Hayes, T. R.; Davis, T. C.; Brooks, J. T.; Claridge, S. A. Plenty of Room at the Top: A Multi-Scale Understanding of nm-Resolution Polymer Patterning on 2D Materials. *Angew. Chem., Int. Ed.* **2021,** *60*, 25436-25444.

18. Shi, A.; Singh, A.; Williams, L. O.; Arango, J. C.; Claridge, S. A. Nanometer-Scale Precision Polymer Patterning of PDMS: Multiscale Insights into Patterning Efficiency Using Alkyldiynamines. *ACS Appl. Mater. Interf.* **2022,** *14*, 22634-22642.

19. Williams, L. O.; Nava, E. K.; Shi, A.; Roberts, T. J.; Davis, C. S.; Claridge, S. A. Designing Interfacial Reactions for Nanometer-Scale Surface Patterning of PDMS with Controlled Elastic Modulus. *ACS Appl. Mater. Interf.* **2022,** *15*, 11360-11368.

20. Okawa, Y.; Aono, M. Linear Chain Polymerization Initiated by a Scanning Tunneling Microscope Tip at Designated Positions. *J. Chem. Phys.* **2001,** *115*, 2317-2322.

21. Espeau, P.; White, J. W. Thermodynamic Properties of N-Alkanes in Porous Graphite. *J. Chem. Soc. - Farad. Trans.* **1997,** *93*, 3197-3200.

22. Espeau, P.; White, J. W. The Phase Transitions of N-Alkanes in Mesoscopic Pores of Graphite. *Carbon* **2005,** *43*, 1885-1890.
